# Genome-wide CRISPR-dCas9 screens in *E*. *coli* identify essential genes and phage host factors

**DOI:** 10.1371/journal.pgen.1007749

**Published:** 2018-11-07

**Authors:** François Rousset, Lun Cui, Elise Siouve, Christophe Becavin, Florence Depardieu, David Bikard

**Affiliations:** 1 Synthetic Biology Group, Microbiology Department, Institut Pasteur, Paris, France; 2 Sorbonne Université, Collège Doctoral, Paris, France; 3 Hub Bioinformatique et Biostatistique, Institut Pasteur - C3BI, USR 3756 IP CNRS, Paris, France; Swiss Federal Institute of Technology Lausanne (EPFL), SWITZERLAND

## Abstract

High-throughput genetic screens are powerful methods to identify genes linked to a given phenotype. The catalytic null mutant of the Cas9 RNA-guided nuclease (dCas9) can be conveniently used to silence genes of interest in a method also known as CRISPRi. Here, we report a genome-wide CRISPR-dCas9 screen using a starting pool of ~ 92,000 sgRNAs which target random positions in the chromosome of *E*. *coli*. To benchmark our method, we first investigate its utility to predict gene essentiality in the genome of *E*. *coli* during growth in rich medium. We could identify 79% of the genes previously reported as essential and demonstrate the non-essentiality of some genes annotated as essential. In addition, we took advantage of the intermediate repression levels obtained when targeting the template strand of genes to show that cells are very sensitive to the expression level of a limited set of essential genes. Our data can be visualized on CRISPRbrowser, a custom web interface available at crispr.pasteur.fr. We then apply the screen to discover *E*. *coli* genes required by phages λ, T4 and 186 to kill their host, highlighting the involvement of diverse host pathways in the infection process of the three tested phages. We also identify colanic acid capsule synthesis as a shared resistance mechanism to all three phages. Finally, using a plasmid packaging system and a transduction assay, we identify genes required for the formation of functional λ capsids, thus covering the entire phage cycle. This study demonstrates the usefulness and convenience of pooled genome-wide CRISPR-dCas9 screens in bacteria and paves the way for their broader use as a powerful tool in bacterial genomics.

## Author summary

Over the past few years, CRISPR-Cas technologies have emerged as powerful tools to edit genomes and modulate gene expression. They have been applied to perform high-throughput genetic screens with the purpose to understand the function of genes in a systematic manner, but the application of these screens to bacteria have so far remained limited. Here, we present the use of a library of ~92,000 guide RNAs directing the dCas9 protein to silence one by one all the genes in the chromosome of *E*. *coli*. To benchmark our method, we first investigate the performance of the technique to identify essential genes, highlighting several non-essential genes also found to be essential by other methods. We then apply our method to detect bacterial genes required by three different bacteriophages to kill *E*. *coli* and for the production of functional progeny by phage λ. Our screens highlight previously known and new genetic interactions between phages and their host’s pathways and emphasize the importance of bacterial capsule in the resistance to multiple phages. Altogether, our results demonstrate the usefulness of genome-wide CRISPR-dCas9 screens in bacteria to uncover genes involved in various phenotypes.

## Introduction

The technological applications of RNA-guided nucleases derived from the Clustered Regularly Interspaced Short Palindromic Repeat (CRISPR) prokaryotic immune system [1–3] represents a true paradigm shift in our ability to manipulate cells at the genetic level [4]. In particular, the Cas9 nuclease from type II systems can be guided by a chimeric single-guide RNA (sgRNA) which directs it to specifically cleave target sequences [5]. The ease with which these tools can be reprogrammed enables the development of powerful high-throughput screens. Recent studies have shown how an engineered CRISPR system packaged in lentiviral vectors can be used to deliver libraries of guide RNAs to human cells and create libraries of gene knockouts [6,7]. Such libraries can target most genes in the genome and were used, among other things, to identify essential genes in the human genome [8–12] and genetic requirements for different human viruses [13–15].

This approach is not directly applicable to bacteria where Cas9 cleavage leads to cell death rather than gene knockout [16–20]. The catalytic dead variant of Cas9, known as dCas9, carries mutations inactivating its two catalytic domains. It binds specific targets without introducing a double-strand break and can conveniently be used to silence genes in bacteria [21,22]. The strand orientation of dCas9 binding does not seem to matter when blocking transcription initiation by targeting a promoter sequence; however binding of the guide RNA to the non-template (coding) strand is required to block the running RNA polymerase.

Arrayed libraries of a few hundreds of guide RNAs have already proven useful to decipher the function of essential genes in *Bacillus subtilis* and *Streptococcus pneumoniae* [23,24]. While arrayed libraries enable to access many phenotypes and easily link them to genotypes, they are cumbersome to work with and maintain. Pooled screens present the major advantage of enabling the study of much larger libraries at a low cost, using well-established high-throughput sequencing methods [8–15,25].

We recently constructed a pooled library of ~ 92,000 sgRNAs targeting random positions in the genome of *E*. *coli* MG1655 with the sole requirement of a proper NGG protospacer adjacent motif (PAM) [26]. This library enabled us to investigate the properties of CRISPR-dCas9 screens in *E*. *coli* in an unbiased way and identify several important design rules to avoid off-target effects and toxicity of dCas9. In this study, we now analyze the results of the screen taking these design rules into account to investigate what it can teach us about gene essentiality in *E*. *coli* during growth in rich medium. We show that this method can be used to confidently predict gene essentiality in most cases despite the polar effect produced by dCas9. Among other findings, we further reveal the importance of some repeated elements, identify essential genes that cannot tolerate small reductions in their expression levels, and challenge the essentiality of a few genes.

As a proof of concept of the usefulness of this approach, we then applied the screen to identify *E*. *coli* genes required for bacteriophage infection. This knowledge can help identify genes that bacteria can mutate to become phage-resistant, an interesting insight for the design of improved phage therapies. More generally, the study of phage-host interactions over the last century has led to many basic discoveries in genetics and molecular biology, as well as to the development of powerful molecular biology tools [27,28]. In addition to classical genetic approaches, genome-wide screens have already been performed to identify host dependencies of *E*. *coli* phages T7 and λ using the Keio collection, an in-frame single-gene knockout strain collection [29–31]. While powerful, this method shows several limitations, including the time-consuming development of such strain collection, the laborious screening process and the focus on nonessential genes only. Here, the application of our CRISPR-dCas9 screen to the study of phages λ, T4 and 186 revealed various host factors including phage receptors and lipopolysaccharide (LPS) requirements. It also highlighted capsule synthesis as a shared resistance mechanism to the three phages. We finally took advantage of the ability of phage λ to package plasmids carrying a *cos* site to perform a pooled transduction assay of the library, enabling to distinguish host genes used by phage λ to lyse its host from host genes required for the production of functional λ particles.

## Results

### Screen design

**Fig 1:**
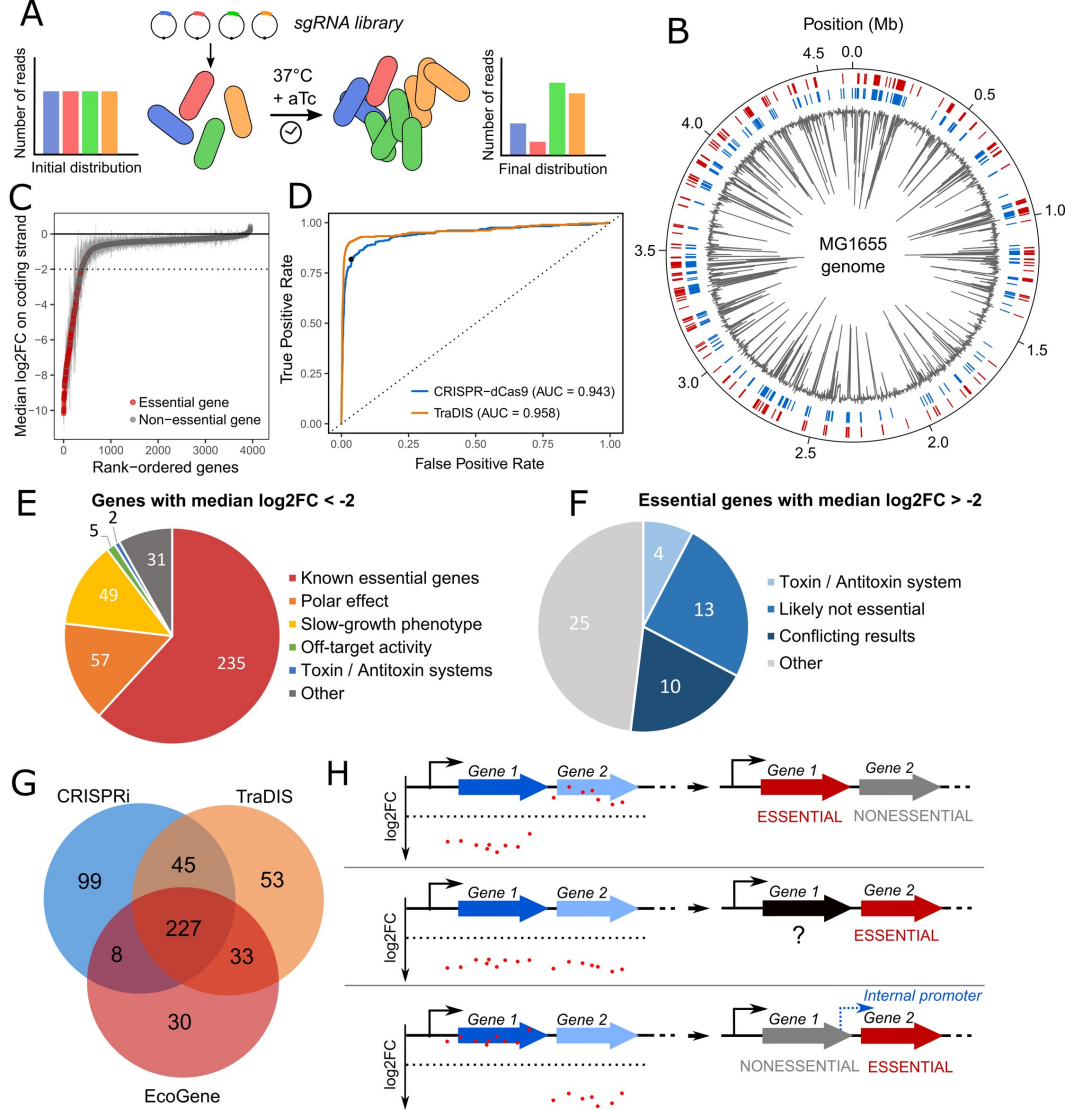
Predicting *E*. *coli* essential genes from the CRISPR-dCas9 screen data. (A) A sgRNA library was transformed into cells expressing dCas9 under the control of an aTc-inducible promoter. The distribution of sgRNAs was retrieved by deep sequencing before dCas9 induction and after 17 generations of induction. (B) Genome-wide visualization of the screen results. The median log2FC of sgRNAs targeting the coding strand of protein-coding genes is displayed in grey. The outer red track represents genes annotated as essential in the EcoGene database while blue track shows genes selected as candidate essential in our screen (median log2FC < -2) (C) Genes were ranked according to the median log2FC of the sgRNAs targeting the coding strand of protein-coding genes. Genes annotated as essential in the EcoGene database are shown in red. Dashed line represents the chosen essentiality threshold (median log2FC = -2). Error bars represent median absolute deviation. (D) The median log2FC of sgRNAs targeting the coding strand of protein-coding genes was used to predict gene essentiality. Receiver Operating Characteristic (ROC) curve of the prediction model is plotted (AUC = 0.943). The black dot represents the chosen threshold. The index score from the TraDIS dataset was also used to predict the EcoGene essential gene set (AUC = 0.957). (E) 379 candidate essential genes were chosen with the threshold. Polar effect indicates genes located upstream essential or near-essential genes. Slow-growth phenotype indicates non-essential genes known to have a growth defect when mutated or deleted. (F) 52 genes annotated as essential were not classified as essential in our screen. Genes found essential only in the Keio collection but not by other sources are termed as “likely not essential”. Conflicting results indicates genes with discrepancies between various datasets. (G) Venn diagram comparing essential genes selected in our screen, in the TraDIS study [35] and in the EcoGene database [34]. (H) Within an operon, different scenarios are displayed as well as the corresponding conclusion. If a fitness defect is induced by guides targeting the first gene but not the second gene, then the first gene can be confidently classified as essential and the second as non-essential (top panel). If a fitness defect is induced when targeting both genes, then the second gene can confidently be classified as essential while the first remains uncertain (middle panel). Finally, if a fitness defect is induced by guides targeting the second gene but not the first gene, then an internal promoter likely drives the expression of the second gene (bottom panel).

In our previous work, we performed CRISPR-dCas9 screens using a library 92,000 guide RNAs grown over 17 generations in rich medium. We constructed to this end strain LC-E75, a derivative of *E*. *coli* MG1655 carrying dCas9 on its chromosome under the control of an aTc-inducible promoter [26]. In this strain, the expression level of dCas9 was fine-tuned to limit a surprising phenomenon that we termed the “bad-seed” effect: dCas9 appears to be toxic to *E*. *coli* when guided by sgRNAs sharing some specific 5 nt PAM-proximal sequences. This effect is particularly pronounced at high dCas9 concentrations and could be alleviated by tuning dCas9 levels while maintaining strong on-target repression. Tuning dCas9 concentration also enabled to limit off-target repression of genes, which we found could occur with as little as 9 nt of identity between the PAM-proximal region of a guide and an off-target. We now analyze the data obtained from this screen to study gene essentiality in *E*. *coli*. During this experiment, guides that reduce the cell fitness for instance by blocking the expression of essential genes are depleted from the library (Fig 1A). The DNA from the sgRNA library was extracted and sequenced before and after dCas9 induction. We used the number of reads as a measure of the abundance of each guide in the library and computed the log2-transformed fold change (log2FC) from DESeq2 R package [32] as a measure of relative sgRNA fitness.

### Effect of guides targeting multiple positions

While most guides in our screen target unique positions in the genome, our library also includes guides targeting multiple positions. These guides can be conveniently used to investigate the role of genes or genomics regions present in several copies in the genome, which cannot be easily achieved by other methods. Interestingly, 18.0% (348/1,932) of sgRNAs with multiple targets around the genome are strongly depleted (log2FC < -2) against 7.4% (6,117/82,816) for guides having a single target (p < 10^−41^, Fischer’s exact test). Most of these depleted guides target genes involved in translation: ribosomal RNAs, tRNAs, and the elongation factor Tu encoded by highly similar *tufA* and *tufB* genes (S1 Fig). More surprisingly, 37 of these guides target Repetitive Extragenic Palindromic (REP) elements. Some REP elements were reported to play a regulatory role at the translational level by modulating mRNA stability [33], but their exact role remains elusive. For many of these guides, none of the target positions are in the vicinity of essential genes, suggesting that the fitness defect caused by dCas9 binding at REP sequences is not simply due to an effect on the expression of essential genes. Guides targeting several repeat regions at the same time had a significantly higher chance of inducing a strong fitness defect (log2FC < -2) than guides targeting only one repeat region (p < 2.10^−5^, Fischer’s exact test). Further work will be required to understand the mechanism responsible for the fitness defect produced by REP-targeting guides, which will likely reveal interesting biology.

### Identification of essential genes

Starting from the initial library of ~ 92,000 sgRNAs targeting random positions along the chromosome of *E*. *coli*, we filtered sgRNAs with either a bad seed effect, an off-target, multiple targets in the genome or a low number of reads (see Methods), yielding a library of ~ 59,000 guides used to perform the analyses below (S1 Table). To investigate the usefulness of such CRISPR-dCas9 screens to identify essential genes, we first ranked genes according to the median log2FC of sgRNAs targeting the coding strand (S2 Table). Mapping our data to the genome of *E*. *coli* highlighted a good concordance with previously known essential genomic regions (Fig 1B). As expected, genes with the most depleted sgRNAs included a large majority of genes annotated as essential in the EcoGene database [34] (Fig 1C). A gene network analysis of the 100 top-scoring genes in our screen highlighted the main essential functions, namely ribosome assembly, peptidoglycan synthesis, DNA replication and transcription, fatty acid metabolism and tRNA metabolism (S2 Fig). We then assessed the ability to predict gene essentiality from the median log2FC value, using the EcoGene database as a gold standard. The receiver operating characteristic (ROC) curve showed an area under the curve (AUC) of 0.943 (Fig 1D). Our CRISPR-dCas9 screen achieved a similar prediction performance as a recent screen based on Transposon-Directed Insertion-site Sequencing (TraDIS) using the largest ever built transposon library (901,383 insertion sites, AUC = 0.957) [35]. This suggests that CRISPR-dCas9 screens can reach the efficacy of transposon-derived methods but with much smaller libraries (~ 15 fold in this study). In particular, during the review process, a new study using genome-wide CRISPRi screening in *E*. *coli* reported similar conclusions by achieving a comparable prediction performance with a rationally designed library of ~ 60,000 sgRNAs, thus highlighting the reproducibility of CRISPRi screens [25].

Candidate essential genes were selected when the median log2FC of sgRNAs targeting their coding strand was lower than -2. This arbitrary threshold enabled to select a consistent number of candidate genes while limiting the selection of false positives (see Fig 1D). We selected 379 genes including 235 genes annotated as essential (62%), representing 79% of the annotated essential genes (Fig 1E). Among the 144 remaining genes, 49 are known to show a slow-growth phenotype when mutated or deleted [36] and can thus be termed as near-essential. The effect of 57/144 genes can be explained by a polar effect leading to the repression of a downstream essential or near-essential gene in the same operon. All in all, polar effects account for a false-positive discovery rate of essential genes of 15%. In addition, 2/144 genes, *hipB* and *ratA* are part of toxin/antitoxin systems. Blocking the expression of *hipB* will also silence the downstream *hipA* toxin. This is expected to be toxic as degradation of HipB by the Lon protease will free HipA [37]. The antitoxin of *ratA* was not described and its regulation is poorly understood but we can make the hypothesis that guides targeting this gene also block the expression of the antitoxin. Among the 36 remaining genes, 5 genes can be explained by off-target effects. When filtering the data to eliminate guides with off-targets, we made a compromise between the number of guides to keep in the analysis and the stringency of the filter. As a result, a few guides with off-target effects remain in the library. Since the median log2FC was used to assess gene essentiality, a gene targeted by only one or two guides might be classified as essential if one of the guides has an off-target. Two such examples are given in S3 Fig. Finally, 31 genes remain ambiguous either because they are targeted by a very low number of sgRNAs in our library or because these genes may be essential or near-essential in our experimental conditions. Note that 7 of these genes (*aceF*, *lpd*, *dcd*, *hemE* and *ihfA*) were found to be essential in the recent TraDIS screen [35]. Overall comparison of our data with the EcoGene database and with the TraDIS screen revealed 227 essential genes common to all datasets while the genes unique to our dataset are mostly explained by polar effects (44/99) and slow-growth phenotypes (25/99) (Fig 1G).

Note that the analysis above was performed by computing a median log2FC value for each gene without taking into account their genomic context. Silencing a gene in an operon with dCas9 leads to the repression of all the downstream genes, and in many cases, several genes with a fitness defect are adjacent within an operon. In this scenario, only the last gene with a fitness defect can confidently be classified as essential or near-essential due to polar effects. Upstream genes might or might not cause a fitness defect when silenced alone, and can be classified as potentially essential (Fig 1H). When doing the analysis in this manner rather than gene by gene, only 155 genes can be confidently classified as essential while 224 genes remain uncertain.

Another interesting scenario when looking at operons is when silencing a gene upstream of an essential or near-essential gene induces no fitness defect despite the theoretical polar effect. A likely explanation is that an unidentified promoter internal to the operon drives the expression of the essential gene independently of the upstream gene (Fig 1H). We identified 7 such cases (S4 Fig), and in all instances recent data supports the existence of an internal promoter [38].

Conversely, some genes annotated as essential do not show a strong fitness defect in our screen (median log2FC > -2) (Fig 1F, S3 Table). Out of 52 essential genes that we fail to identify, 4 genes (*chpS*, *mazE*, *yafN* and *yefM*) are part of toxin-antitoxin systems which are encoded in operons with the antitoxin gene preceding the toxin gene. By definition, an antitoxin is essential to the cell as silencing it leads the cognate toxin to kill the cell. In the four cases above, dCas9 blocks the expression of both the antitoxin and the toxin gene, which we expected to be toxic if the antitoxin is more labile. Surprisingly, unlike *hipB*, no effect on fitness was measured here. Another 13/52 genes annotated as essential in the Ecogene database were also supposed to be non-essential by other studies [35,39,40]. We successfully replaced three of these genes, *alsK*, *bcsB* and *entD* by a kanamycin resistance cassette, confirming that they are indeed not essential (S5 Fig). In 10/53 other cases, gene essentiality has also been challenged by conflicting results [31,35,39–41]. For the remaining 25 genes, the absence of fitness defect could be explained by an inefficient repression by dCas9, a strong robustness of the cell to low levels of the protein or could indicate genes that are actually not essential in our experimental conditions. In addition, weak repression could arise from negative regulatory feedback loops. We recently showed that silencing a gene regulated by a negative feedback leads to an increased transcription initiation rate, ultimately leading to weak silencing [42]. Here, we specifically investigated two essential genes, *lexA* and *rho*, which are not detected as essential in our screen and whose expression is known to be controlled by a negative feedback loop: *lexA* binds its own promoter while the expression of *rho* is attenuated by the presence of Rho-dependent terminators when Rho levels are high. As expected, targeting these genes only led to a weak repression level as measured by RT-qPCR (S6 Fig). When cloning a sfGFP reporter under the control of the *lexA* promoter or the *rho* leader sequence, we could confirm that blocking *lexA* or *rho* led to an increased sfGFP expression (S6 Fig). We can make the prediction that guide RNAs binding in the promoter region of *lexA* should not be affected by the negative feedback loop since they inhibit the initiation of transcription. Indeed, a guide targeting the promoter of *lexA* does show a strong fitness defect (no guide targeting the promoter of *rho* are present in the screen).

### Unexpected effects on the template strand of target genes

**Fig 2:**
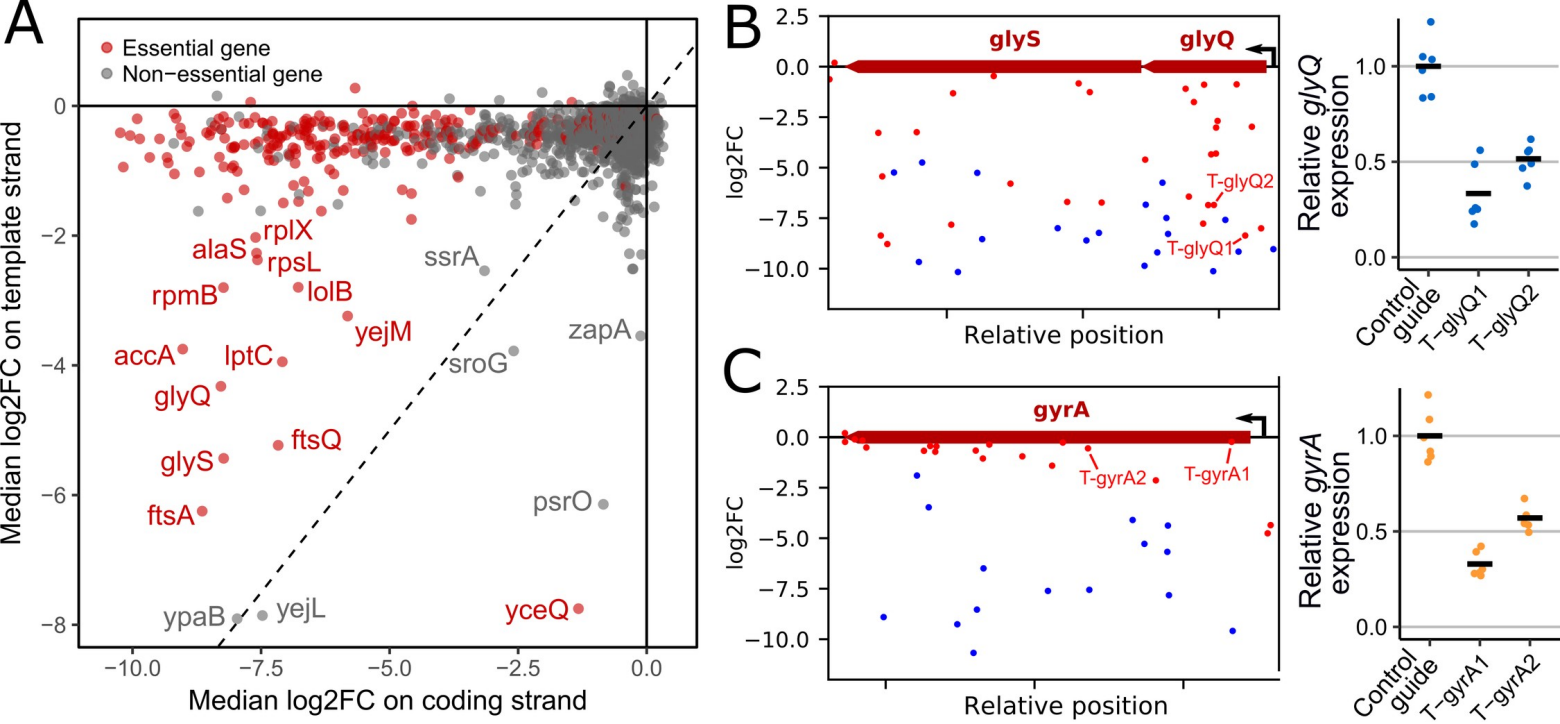
Identification of essential genes that do not tolerate small changes in their expression level. (A) Comparison between median log2FoldChange of sgRNAs targeting the coding strand or the template strand highlights unexpected effects. (B) Effect of guide RNAs targeting the *glyQS* operon. (C) Effect of guide RNAs targeting *gyrA*. (B,C) Relative expression of *glyQS* and *gyrA* when targeted by guides T-glyQ1, T-glyQ2, T-gyrA1 and T-gyrA2 as measured by RT-qPCR. Black bars show the mean of 3 independent biological replicates and 2 technical replicates.

We further investigated the effect of sgRNAs targeting the template strand of genes. Guides in this orientation should only have a moderate effect on gene expression. As expected, the vast majority of them do not produce any fitness defect. We compared the median log2FC of sgRNAs targeting the coding strand and the template strand (Fig 2A). Interestingly, we observed that a set of essential genes that we term “sensitive” produced a strong fitness defect (median log2FC < -2) when targeted on the template strand (including *accA*, *alaS*, *ftsA*, *ftsQ*, *glyQ*, *glyS*, *lolB*, *lptC*, *rplX*, *rpmB*, *rpsL* and *yejM*) whereas targeting the template strand of other essential genes did not induce any fitness defect. To verify that this effect does not occur from a surprisingly high repression level on the template strand of these genes, we measured the repression efficiency of 2 guides targeting the template strand of the “sensitive” gene *glyQ* (Fig 2B). As a control, we also measured the repression efficiency of 2 guides targeting the template strand of the essential gene *gyrA* which does not show this sensitivity phenotype (Fig 2C). The results show similar intermediate repression levels in both cases (33–57% of the initial expression level, see Fig 2B and 2C) suggesting that the cell indeed seems very sensitive to the expression level of some essential genes whereas a weak repression of most essential genes can be tolerated.

The other genes that display a strong fitness defect when targeted on the template strand can be explained by polar effects on nearby essential or near-essential genes and shed light on atypical gene organizations (S7 Fig). Gene *yejL* is located upstream of the sensitive essential gene *yejM*. Gene *ypaB* contains the promoter of the essential gene *nrdA*. Gene *sroG* is actually a riboswitch controlling the expression of the essential gene *ribB* involved in riboflavin synthesis [43]. One sgRNA binding to the template strand in the very beginning of the gene was strongly depleted, suggesting that it blocks transcription initiation of the *ribB* mRNA. *yceQ* is a small open reading frame of just 321bp in the promoter region of the essential RNAse E gene (*rne*) and in the opposite orientation. Guide RNAs directing dCas9 to bind the template strand of *yceQ* are thus in the good orientation to effectively block the expression of *rne*. Conversely, guides targeting the coding strand of *yceQ* show no fitness defect, suggesting that *yceQ* is actually not essential, which is also supported by recent TraDIS data [35]. A similar case is that of *psrO* which encodes a small RNA located in the promoter region of *pnp*.

Overall, these results show the performance of CRISPRi screens to assess gene essentiality in *E*. *coli* but also to highlight diverse genomic organizations. Our results can be visualized on CRISPRbrowser, a web-based genome browser available at crispr.pasteur.fr.

### Identification of genes providing phage resistance when silenced

To demonstrate the broad usefulness of this strategy to the study of different phenotypes, we applied the method to unveil bacterial genes required for successful phage infection, also known as host factors. Considering previous results, we now focused on sgRNAs targeting genes on the coding strand, yielding a library of ~ 17,220 sgRNAs after removing guides with an insufficient number of reads. As a proof of concept, we used temperate phage λ whose host requirements are well documented. We also used temperate phage 186cIts (a thermosensitive strain of phage 186) and virulent phage T4 in order to compare their host requirements. As strain LC-E75 carries the *dcas9* expression cassette integrated at the *attB* site of phage 186, we constructed a new strain, FR-E01, with the same cassette integrated at the *attB* site of phage HK022 in order to avoid any interference with phage 186. Both strains expressed dCas9 at the same level (S8 Fig).

**Fig 3:**
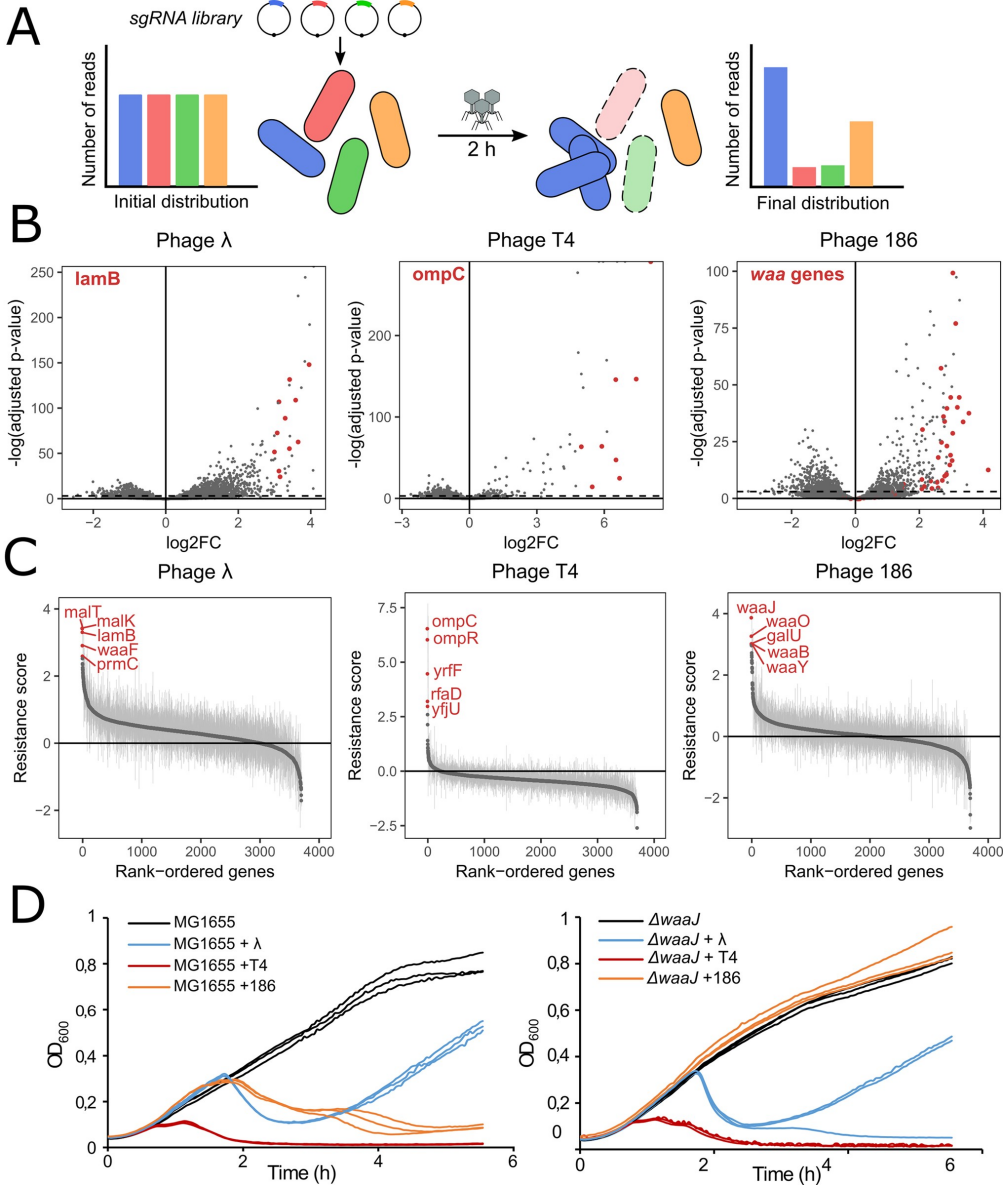
The CRISPRi screen reveals phage receptors and LPS requirements of phages λ, T4 and 186. (A) Overview of the experimental procedure. dCas9 expression was induced for ~ 8h before infection by λ, T4 or 186 at a MOI of 1. The distribution of sgRNAs was retrieved before and after infection by sequencing. (B) The log2FoldChange was computed for each sgRNA. For phages λ and T4, sgRNAs targeting the phage receptor (respectively LamB and OmpC) are highlighted in red as well as genes targeting *waa* genes for phage 186. Dashed line represents adjusted p-value = 10^−3^. (C) For each gene, a resistance score was computed as the median log2FoldChange of sgRNAs targeting the coding strand. Error bars represent median absolute deviation. 5 top-scoring genes are highlighted in red in each panel. *(D)* Infection time courses of MG1655 (top panel) or Keio collection strain *ΔwaaJ* without phage or after infection by λ, T4 or 186 (MOI ~ 0.01).

A culture of strain FR-E01 carrying the library was grown with aTc to stationary phase allowing for dCas9 to be expressed and for the target gene products to be diluted and/or degraded. Cells were then diluted 100-fold and grown to exponential phase, still with aTc, followed by infection with phage λ, T4 or 186cIts at a multiplicity of infection (MOI) of 1 (Fig 3A). During the experiment, phages will lyse bacteria unless the sgRNA they carry makes them resistant to infection. The pool of sgRNAs was sequenced before and after 2 h of infection to allow phages to lyse a maximum of sensitive cells while limiting the rise of resistant mutants in the population. Log2FC values were computed for each sgRNA as previously (S4 Table and Fig 3B). For each gene, a resistance score was computed as the median log2FC of the guides targeting the coding strand (S5 Table).

Host genes are required at several steps of the phage infectious cycle and the genes involved differ depending on each phage. The first step of phage infection is adsorption to the cell followed by receptor binding and injection of the phage DNA. Surface proteins or specific structures of the LPS are typically used as receptors. The gpJ tail tip protein of phage λ binds to the maltose outer membrane porin LamB [44], while T4 recognizes both the OmpC protein and the core-lipid A region of the LPS that includes the heptose region [45]. In addition to surface receptors, in the case of λ, the ManXYZ mannose permease helps the phage DNA cross the inner membrane [46]. The surface receptor of phage 186 or other genes required for DNA entry are not known. As expected, the highest-scoring genes in our screen correspond to phage receptors and their positive regulators (Fig 3B and 3C). The phage λ receptor gene *lamB* and its regulators *malT*, *cyaA* and *crp* respectively ranked 3^nd^, 2^st^, 6^th^ and 9^th^ after infection by λ. The gene with the 1^st^ highest resistance score was *malK* which is expressed upstream of *lamB* in an operon structure. Guides that block the expression of this gene therefore also block the expression of *lamB*, explaining its high ranking although it is not known to participate in the infection process. Many genes involved in LPS biosynthesis (*lpcA*, *gmhB*, *rfaD*, *rfaE*, *waaA*, *waaC*, *waaF*, *waaG)* were also identified in our screen. Mutations affecting LPS synthesis were previously associated with reduced adsorption of λ, presumably linked to a reduced availability of LamB on the outer membrane [47]. Guides that target the manXYZ operon where also significantly albeit weakly enriched (p < 0.0034, Mann-Whitney *U* test). In the case of phage T4, gene *ompC* and its regulator *ompR* ranked 1^st^ and 2^nd^ while genes *rfaD* and *waaF* involved in the synthesis of glycerol-D-manno-heptose and its addition to the LPS, ranked 5^th^ and 8^th^. Considering that the receptor for phage 186 is not clearly established, we used this approach to identify candidate host components. Out of the 20 highest scoring genes, 16 were involved in LPS biosynthesis while no surface proteins were present, suggesting that 186 uses the LPS as a receptor (Fig 3C). To validate this observation, we performed infection time courses with a strain deleted for *waaJ*, one of the last genes in the LPS biosynthesis pathway, in the presence of each of the three phages. The absence of gene *waaJ* induced a strong resistance to 186 but not to λ and T4 (Fig 3D) suggesting that the outer core of the K-12 strain LPS is required for adhesion of phage 186. Taken together, these observations validate the ability of our method to identify phage receptors which are the most common bacterial components giving rise to phage resistance.

**Fig 4:**
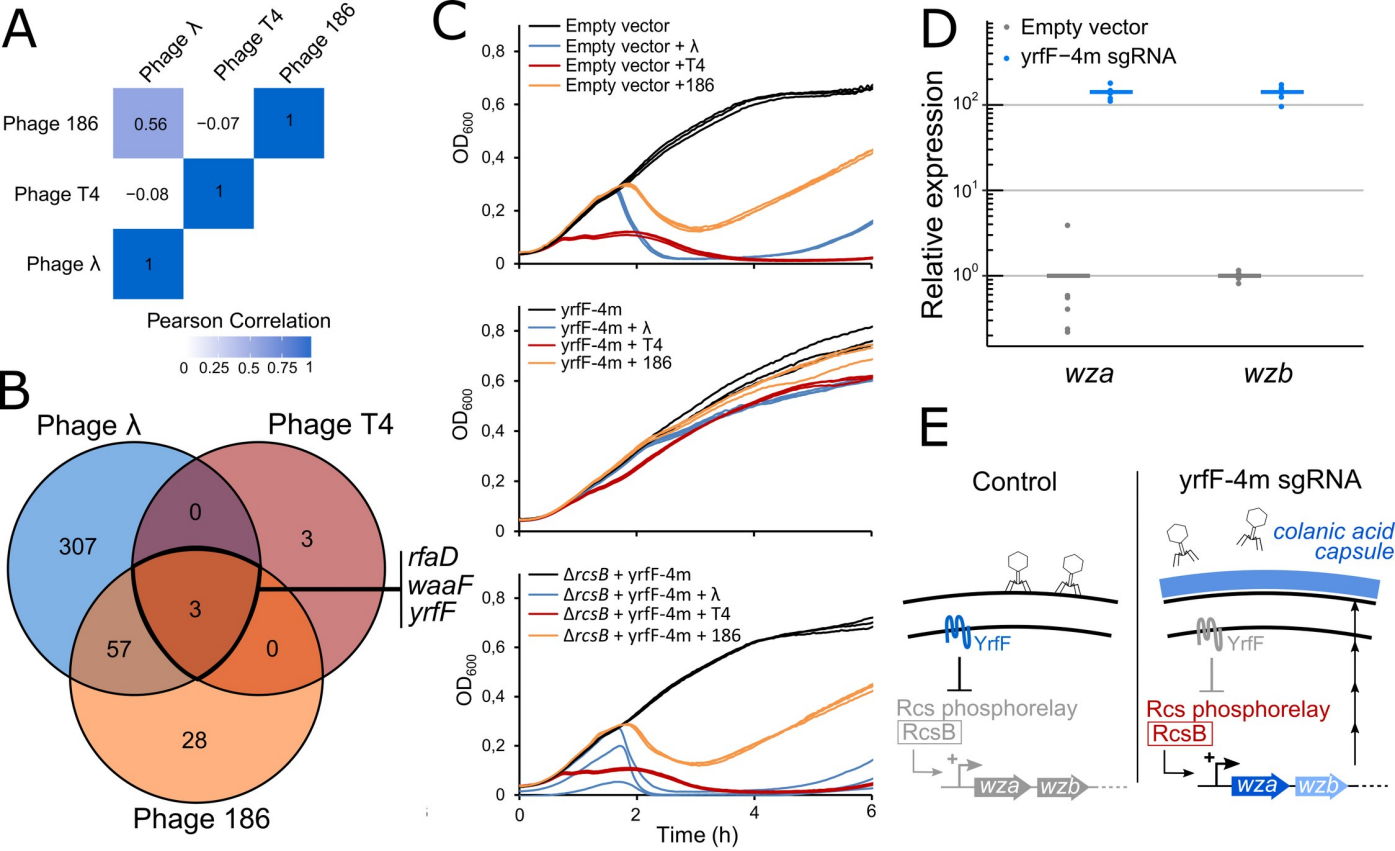
Colanic acid capsule synthesis is a common resistance mechanism to phages λ, T4 and 186. (A) Pearson correlation coefficient of resistance scores between phages λ, T4 and 186-cIts. (B) For each phage, genes targeted by > 1 guide with a resistance score greater than 20% of the maximum score were selected and compared in a Venn diagram. (C) Infection time courses of FR-E01 carrying an empty vector, FR-E01 expressing *yrfF* sgRNA with 4 mismatches (yrfF-4m) or FR-E01Δ*rcsB* expressing yrfF-4m without infection or after infection by λ, T4 or 186-cI (MOI ~ 0.01). Experiments were performed in triplicates. (D) Expression of genes *wza* and *wzb* involved in colanic acid capsule synthesis in strain FR-E01 carrying an empty vector or expressing the yrfF-4m sgRNA, as measured by RT-qPCR. Results are shown for 3 biological replicates and 2 technical replicates. (E) Schematic of colanic acid capsule-mediated phage resistance. YrfF inhibits the Rcs phosphorelay. Inhibition of YrfF expression activates the Rcs phosphorelay which triggers the expression of genes involved in colanic acid capsule synthesis.

Comparison between phages λ, T4 and 186 revealed closer host requirements between the two temperate phages, λ and 186, than between λ and T4 or between 186 and T4 (Fig 4A). Genes whose silencing provide resistance to the different phages were selected if their resistance score was at least 20% of the maximum score for each phage (see Methods, Fig 4B). Interestingly, we could identify 3 genes with a high resistance score to all 3 phages. These include *rfaD* and *waaF* discussed above for their involvement in LPS biosynthesis. In addition, we identified gene *yrfF*, an essential gene indirectly linked to capsule synthesis. YrfF inhibits the Rcs signaling pathway by an unknown mechanism [48]. This signaling pathway senses and responds to damage to the membrane or to the peptidoglycan by activating genes involved in colanic acid capsule synthesis [49,50]. We hypothesized that silencing *yrfF* would lead to the activation of the Rcs signaling pathway, induce capsule synthesis and prevent phage adsorption to the cell surface. The synthesis of capsule was indeed previously reported to provide broad resistance to phages [51–54]. Since *yrfF* gene is essential, we reduced its expression to intermediate levels using a sgRNA bearing 4 mismatches at the 5’-end (yrfF-4m) instead of using a fully matched sgRNA [21,42]. The resulting strain only showed a slight growth defect and became resistant to all three phages (Fig 4C). Deletion of gene *rcsB*, a central actor of the Rcs pathway, restored sensitivity to the 3 phages, showing that the resistance phenotype that results from *yrfF* silencing is mediated by the Rcs pathway. We further confirmed this by showing that silencing *yrfF* induces a ~ 140-fold increase in the expression of the *wza-wzb* operon directly involved in colanic acid capsule synthesis (Fig 4D and 4E). Interestingly, deletion of *rfaD* or *waaF* (but not other genes of the LPS biosynthesis pathway) was shown to induce a mucoid phenotype through the synthesis of a capsule [55]. This suggests that they might provide resistance to all three phages through this pathway rather than through their role in LPS synthesis. Consistently with this hypothesis, silencing genes upstream of *rfaD* and *waaF* in the LPS biosynthesis pathway did not provide resistance to T4 in our screen.

After DNA entry, temperate phages make a decision to either enter the lytic or lysogenic cycle. In the lysogenic cycle, the phage integrates in the host genome and the cell survives. Host factors that increase the rate of lysogeny when repressed are thus expected to be enriched in our screen. As a matter of fact, we identify genes *hflD* and *ftsH* (*hflB*) after λ infection. These genes were historically described through the identification of mutants that affect the lysis/lysogeny decision of λ (*hfl* stands for “high-frequency of lysogeny”) [56]. HflD is known to directly interact with the transcription factor CII of λ and facilitate its degradation by the FtsH-HflKC protease [57]. Repressing *hflD* or *ftsH* is thus expected to increase the level of CII which will push the phage toward lysogeny and increase survival. Genes *hflKC* did not pass our selection threshold for an unknown reason. Interestingly, *hflD* is also found for phage 186 suggesting a similar control of the lysogeny decision for both phages.

In lytic cycle, phages replicate their DNA to high copy number and produce the phage capsids into which the DNA is packaged. Finally, the cell is lysed leading to phage release. Host genes that are required for any of the steps of the lytic cycle are expected to be enriched in our screen as long as silencing their expression prevents cell death. After λ infection, we could identify genes *dnaKJ* encoding a chaperone known to be required for DNA replication of λ [58]. These genes are also required for phage 186 which suggests a similar replication mechanism. Genes involved in two tRNA modification pathways were also identified after λ infection: uridine 2-thiolation (*tusA*, *tusBCD*, *tusE* and *mnmA*) and the essential N^6^-threonylcarbamoyladenosine modification (*tsaB*, *tsaC*, *tsaD* and *tsaE*). Uridine 2-thiolation has been previously described to be necessary for the proper translation of λ proteins gpG and gpGT involved in tail assembly through its impact on programmed ribosomal slippage [29,59], but the essential N^6^-threonylcarbamoyladenosine modification of tRNAs was not previously associated with λ infection. We also identified RNAses *rnt*, *rnpA* and *rnd* involved in tRNA maturation. Altogether, 27 out the 100 top-scoring genes after λ infection are involved in translation either directly or through ribosome or tRNA synthesis. These include genes also found after infection by phage 186 such as *deaD*, *hpf*, *prfA*, *prmC*, *rluD*, *rnpA*, *tilS* and *trmH*. This highlights the importance of an intact translation machinery for the completion of the lytic cycle for both λ and 186. Finally, genes linked to DNA repair including *recA*, *recBCD*, *recG*, and *ligA* seem to be important for 186 infection but not for λ infection. To our knowledge, no recombinase has been identified in the genome of phage 186 suggesting that it may rely on the host recombination system while phage λ carries its own recombination machinery (*gam*, *exo* and *bet*) [60].

### Identification of genes involved in later steps of λ infection

**Fig 5:**
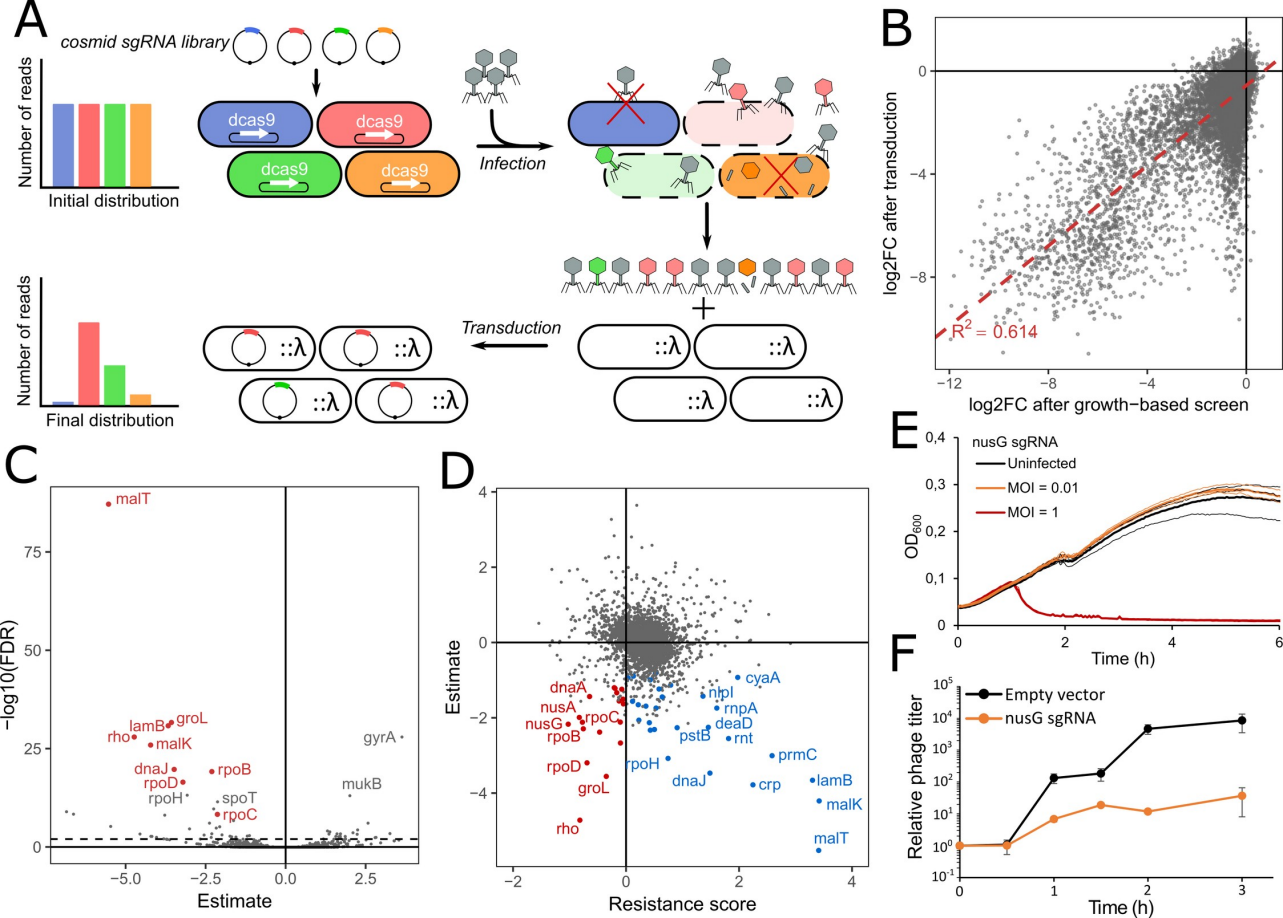
Transduction of the library reveals host genes necessary for the production of functional capsids. (A) Overview of the experimental system to screen for λ host factors. Strain FR-E01 expressing dCas9 under aTc-inducible promoter and carrying the sgRNA library on a cosmid was infected by λ. The library can be packaged into capsids when the sgRNA does not disrupt phage propagation. After transduction into a lysogenic strain, the distribution of sgRNAs is measured by sequencing. (B) log2FC values after transduction are strongly correlated to the effect of guides on cell growth (ANOVA, F = 27263, df = 17198, p < 10^−16^). (C) For each gene, a linear model was built to explain log2FoldChange after transduction while taking the effect on cell growth into account. Estimate and corrected p-value (FDR) are shown for each gene. Genes previously described as host factors are shown in red. (D) Comparison of results after infection and after transduction. Genes targeted by >1 guide which significantly decrease the production of functional phages (ANOVA, FDR < 0.05) are displayed in red or blue when they show a negative or positive resistance score respectively, as computed in the first screen. (E) Growth of strain FR-E01 carrying a sgRNA targeting *nusG* without infection or after infection by λ at MOI = 0.01 or MOI = 1. (F) Phage titer during infection of strain FR-E01 carrying an empty vector or expressing a sgRNA targeting *nusG* (MOI = 0.01). Error bars show standard deviation of three independent experiments.

The screen performed here is thus a powerful method to identify genes required by phages to kill the cell. However, this first strategy cannot identify genes necessary for the synthesis of functional phage capsids if blocking the expression of these genes does not prevent the phage from killing the cell. One can expect that this will be the case of any host gene involved in late stages of the infectious cycle when the host cell is already doomed. In order to get better insights into the genes affecting the production of functional phages, we implemented a second step focusing on phage λ (Fig 5A). The vector carrying the sgRNA library was designed to carry a λ packaging site (*cos* site), thus forming a cosmid [61]. Upon infection, phage λ is thus able to package the plasmid carrying the guide RNA only if a functional capsid was produced (S9 Fig). After infection, the cell lysate containing a mixture of λ and cosmid particles was used to transduce strain MG1655::λ and thus recover guides in the library that do not affect the infection process. The distribution of sgRNAs recovered after transduction was compared to the initial pool to identify depleted sgRNAs corresponding to bacterial genes required for the production of functional capsids (S6 Table).

Interestingly, 45 guides in our library which target prophages of *E*. *coli* MG1655 also have a match with 100% nucleotide identity and a proper PAM sequence in the genome of phage λ. Among these guides, those predicted to block the expression of the structural genes of phage λ should inhibit the production of functional capsids. As expected, these guides were strongly depleted after transduction (S10 Fig). Most of these guides do not block the expression of the lysis genes of λ, explaining why they were not identified in our first screen.

When comparing our data with the essentiality data from our previous screen, we observed a strong correlation between log2FC after transduction and the effect of guides on the cell fitness (Fig 5B). One might indeed expect that genes whose repression slows down cell growth will also slow down phage production, leading to fewer particles being released after 2h of infection. In order to identify genes which disproportionately affect phage production over cell growth, we performed statistical analyses taking the effect on growth into account (see Methods, S7 Table). We identified 57 genes (46 targeted by > 1 guide) which significantly decrease the amount of transduced particles when repressed (FDR < 0.05) (Fig 5C). These genes could be separated into two groups according to their resistance score (Fig 5D): genes with a positive resistance score provided resistance to lysis when silenced, while genes with a negative resistance score led to an increased sensitivity to the phage but a reduced production of functional phage particles. The first group includes genes involved in the expression of LamB (*lamB*, *malK*, *malT*, *crp*, *cyaA*) as well as other genes identified in the first step, such as *rpoH*, *dnaJ*, *prmC*, *rnt* and *rnpA*. The second group includes genes that could not be identified in the first step of our screen based on their resistance score as they decrease or have little effect on cell survival to λ infection. These genes include well-characterized genes involved in the transcription of the λ genome: RNA polymerase subunits β *rpoB* and β’ *rpoC*, RNA polymerase σ^70^ factor *rpoD* and transcription termination factors *rho*, *nusA* and *nusG* [62–65]. These factors interact with λ protein N to avoid transcription termination at terminator sites present on its genome, thus allowing the expression of downstream genes in a process called transcription antitermination [63]. The identification of *rho* is somewhat surprising. Indeed, while it was found to be associated to the antitermination complex [66], it was not previously described as essential for phage λ transcription. While antitermination at Rho-dependent terminators has been extensively studied, note that *rho* is an essential protein and λ replication was never tested in a null mutant of *rho* to our knowledge.

The screen also identified *groL* encoding the GroEL chaperonin involved in the assembly of the λ head [67–69]. Its transcription is controlled by the heat shock-specific RNA polymerase σ^32^ factor *rpoH* found in the first group [70]. Genes with little effect on survival after λ infection include DNA polymerase III subunit α encoded by *dnaE*, as well as many genes encoding aminoacyl-tRNA synthetases, namely *argS*, *pheS*, *pheT*, *leuS*, *hisS*, *proS*, *trpS*, *valS*, *cysS*, and *metG*, suggesting that these essential elements are a limiting resource for the production of phage particles.

This analysis highlights the fact that silencing some genes inhibits the formation of phage particles while leaving the cell susceptible to lysis. To confirm this observation, infection time courses and phage titer measurements were performed in strain FR-E01 expressing a sgRNA targeting *nusG* which has a negative resistance score (Fig 5E). Infection at a MOI of 1 led to a complete lysis of the cell population showing that silencing *nusG* does not provide resistance to infection. However, infection at MOI = 0.01 did not lead to lysis of the population, presumably because the first infected cells did not release enough active phages to lyse the rest of the population. Measuring phage concentration during infection indeed showed a ~ 10-fold reduction in burst size when *nusG* is silenced (Fig 5F).

## Discussion

This work establishes a proof of concept of the performance of genome-wide CRISPRi screens in bacteria for the identification of essential genes and phage host factors. During the review process, another study reported the use of a library of ~60,000 guides (~15 guides per gene) to identify essential genes in *E*. *coli* [25]. In contrast, our library was designed to target ~92,000 randomly chosen positions with a proper PAM along the chromosome of *E*. *coli* in order to investigate the properties of CRISPRi in an unbiased way. A previous screen performed with this library enabled to identify an intriguing toxicity of dCas9 mediated by 5 nucleotides in the seed sequence of the guide RNA, as well as the existence of off-target effects with as little as 9nt of identity between the guide and the off-target position [26]. After filtering our guides to avoid these effects, ~23,000 guides targeting the coding strand of genes were included in our analyses. Despite the much lower effective size of our library compared to the recently published study, both libraries achieved similar results. This highlights the importance of the design rules established in our previous work. A current limitation of CRISPRi is the heterogeneity in sgRNA repression efficiency. Efforts have been made in Eukaryotic systems to predict sgRNA activity [71–75] but such models remain to be developed in bacteria. The ability to confidently design efficient guides will in the future enable to decrease the necessary library size even more, thus increasing the number of experiments that can be performed simultaneously while decreasing DNA synthesis and sequencing costs.

A notable feature to consider during CRISPR-dCas9 experiments is that when series of genes in operons are found to have an effect, it is theoretically only possible to conclude that the last gene in the series is essential. A gene by gene analysis of our data was nonetheless able to reliably predict gene essentiality with a false positive discovery rate due to polar effects of only 15%. This surprisingly accurate performance is due to the fact that essential genes tend to cluster in the same operons. Yet, this is not always the case and polar effects represent a limitation of what can be done with CRISPRi screens: one can easily identify operons of interest but a detailed understanding of the involvement of each gene requires further investigation with other techniques.

Overall, CRISPRi screens present several key advantages: (i) The expression of dCas9 is inducible, which enables to maintain guides targeting essential genes in the library, allowing their study; (ii) Duplicated regions can easily be studied since sgRNAs can be designed to target identical sequences present in several copies along the genome; (iii) Intermediate repression levels can be obtained either by targeting the template strand of genes or by using sgRNAs with a variable number of mismatches [21,42]; (iv) libraries can be rationally designed to target specific locations or subsets of genes, as opposed to the random insertion of transposons. (v) Preparing libraries for sequencing only requires a simple PCR reaction to amplify the guide RNAs.

In this study, we used these key advantages to investigate essential genes and other interesting features of the *E*. *coli* genome. Overall, 79% (235/298) of the genes previously annotated as essential were correctly identified. A large part of the genes that we failed to identify might not actually be essential. Indeed, our screen showed some discrepancies with annotated databases, particularly with the Keio collection, which were also observed in the other recent CRISPRi screen [25]. We experimentally confirmed that some genes were wrongly annotated as essential, corroborating other recent work [35]. Nonetheless, our screen failed to identify a few well-known essential genes. This might be due to a strong robustness of the cell to low levels of some essential proteins or to a weak dCas9 repression caused by negative feedback loops as we could indeed demonstrate in the case of genes *lexA* and *rho*. Note that the design of our library being random, some genes are only targeted by a small number of guides in our screen, reducing the likelihood to correctly identify them. This will easily be corrected in future screens using rationally designed libraries.

In addition to the investigation of gene essentiality, our results highlighted the importance of REP elements and the usefulness of CRISPRi to investigate such repeated loci. We could also identify genes that cannot tolerate small reductions of their expression level. Proteins encoded by these genes could be promising antibiotic targets as a partial inhibition of their activity is likely lethal. Our screen also highlighted some atypical genomic organizations such as internal promoters within operons or nonessential genes antisense to neighboring essential genes.

It is interesting to discuss the advantages and drawbacks of CRISPR-dCas9 screens in comparison with other genome-wide approaches. Arrayed collections of gene deletions have proven extremely useful for the study of the few bacterial species where they have been constructed. The *E*. *coli* Keio collection [31,41] is used as a reference for the classification of *E*. *coli* genes as essential, and has been employed in many different types of screens, including for the identification of phage host factors [29,30]. However, the development of such strain collections and the screening process are laborious and focused on nonessential genes only. The Keio collection is also known to contain a few errors arising from the failure to obtain some gene knockouts for other reasons than gene essentiality, or from gene duplications occurring during the construction of the collection [41]. Transposon-based strategies such as Tn-seq and TraDIS are powerful methods to identify essential and conditionally-essential genes in a wide range of bacteria [35,76,77] by looking for genes in which no transposition events are recovered. With these approaches, false negatives may arise when the insertion of the transposon does not disrupt the functionality of the protein, or when only a small region of a gene is essential. False positives might also arise from biases in the number of transposon insertions along the genome. Several data analysis pipelines have been developed to take these effects into account [78–80], but recent results still highlighted the need to combine statistical analysis with manual curation in transposon-based screens [35]. Finally, both methods can cause polar effects on the expression of adjacent genes which can be hard to predict as opposed to the predictable effects of dCas9 binding on the expression of downstream genes.

The application of our CRISPRi screen to identify *E*. *coli* genes required for phage infection enabled us to identify well known host factors while providing novel insights into phage-host interactions. Among the highlighted host factors, phage receptors and other genes involved in phage adhesion had the strongest effect. Interestingly, some host factors were common between λ and 186 suggesting some similarities between the infectious cycles of these two temperate phages. Phage T4 relies on very few host components in comparison to λ and 186. This is consistent with the fact that T4 has a much larger genome (169 kbp) than the two other phages (48kbp for λ and 31kbp for 186) and encodes most of the functions it needs to complete its cycle [81]. *E*. *coli* was reported to become resistant to phage T4 only by acquiring mutations that block its adsorption [82,83], whereas *E*. *coli* can also become resistant to λ by acquiring mutations in intracellular components [64]. A TraDIS screen was recently performed in *E*. *coli* O157 to investigate genes involved in susceptibility and resistance to phage T4 [82]. This screen identified a few host factors putatively involved in DNA entry that were not found in the CRISPR-dCas9 screen, suggesting the existence of experiment or strain-specific effects. Note that T4 is known to degrade the host DNA [84]. As a consequence, pooled screens might not be able to identify host factors that act at late stages of the infection as the guide RNA or transposons inserted in the genome will be degraded. Note also that our screen failed to identify a few previously described host factors for λ infection [29], likely for the same reasons that we failed to identify a few essential genes.

A notable result of our screen is the identification of colanic acid capsule synthesis as a shared resistance mechanism to phages λ, 186 and T4. The capsule of *E*. *coli* has been shown to protect against external aggressions such as antibiotics or desiccation and helps to evade the host immune system [51]. While previous studies have reported that bacterial capsule can also provide resistance to several phages [52–54], this is rarely seen as the main function of the capsule. Our findings argue for a broader role of colanic acid capsule synthesis as a defense mechanism against a wide range of phages, acting as a physical barrier by masking phage receptors on the cell surface. Accordingly, some phages have evolved the capacity to degrade capsule through the action of enzymes such as endosialidases which are frequently carried by the tail fibers of caudovirales [52,85–87].

While genome-wide CRISPR screens have already demonstrated their usefulness in eukaryotic systems [8–15], this study should now set the stage for their broader adoption as a powerful tool in bacterial genomics.

## Methods

### Bacterial strains and media

*E*. *coli* strains were grown in Luria-Bertani (LB) broth. LB-Agar 1.5% was used as a solid medium. Antibiotics were used in standard concentrations (50 μg / mL kanamycin (Sigma), 100 μg / mL carbenicillin (Euromedex)). *E*. *coli* DH5α (New England Biolabs) was used as a cloning strain and *E*. *coli* K12 MG1655, a gift from Didier Mazel, was used for screening experiments.

### Phage strains and stocks

The MG1655::λ lysogeny was a gift from Luciano Marraffini, the MG1655::186cI-ts lysogen was a gift from Keith Shearwin and phage T4 was a gift from Laurent Debarbieux. Overnight cultures of MG1655::λ and C600::186cIts were harvested and the supernatant was filtered (0.22 μm). All the liquid stocks were further propagated in MG1655 grown in LB supplemented with maltose 0.2% (Sigma) and CaCl_2_ 5 mM (Sigma) at a multiplicity of infection (MOI) of 1. Phage titers were measured by spotting 2 μL-drops of 10-fold serial dilutions in λ dilution buffer (TrisHCl 20 mM, NaCl 0.1M and MgSO_4_ 10 mM) on a bacterial lawn of *E*. *coli* MG1655 in LB-agar (0.5%) containing 5 mM CaCl_2_.

### *E*. *coli* strain construction

Strain LC-E75 used for the essentiality screen was described in our previous study [26]. This strain expresses an optimized *dcas9* cassette under the control of an aTc-inducible pTet promoter integrated at the phage 186 *attB* site. In order to study phage 186 with our screen, a new strain FR-E01 was constructed with the same cassette integrated at the HK022 *attB* site to avoid any interference. A high-copy pOSIP backbone [88] containing the HK022 integrase, HK022 *attB* site and a Kanamycin-resistance cassette was digested by EcoRI and PstI (New England Biolabs). A fragment containing *dcas9* under the control of pTet promoter was amplified by Phusion PCR (ThermoScientific) with primers LC124/LC125 to introduce homology regions with the backbone. The 2 fragments were assembled by the Gibson method [89]. The resulting vector was electroporated into strain MG1655. The backbone containing the HK022 integrase and a Kan^R^ selection marker was then removed using the pE-FLP (Amp^R^) plasmid able to recombine FRT sites flanking the backbone [88]. pE-FLP was then cured through serial restreaks on LB plates, yielding strain FR-E01. Primers used for cloning are listed in S8 Table. Strain LC-E75 and FR-E01 are available on AddGene with the accession numbers 115925 and 118727 respectively.

### CRISPRi library design and assembly

A library of ~ 92,000 sgRNAs was designed previously [26]. These sgRNAs target 20-nt regions adjacent to NGG sites in *E*. *coli* K-12 MG1655 (NC_000913.2) and were chosen randomly among the total pool of possible sgRNAs in this strain. Briefly, the library was generated through on-chip oligo synthesis (CustomArray), amplified with primers LC296 and LC297 using Phusion DNA polymerase (ThermoScientific) and assembled into the psgRNAcos backbone using the Gibson method [89]. The resulting plasmid library DNA was transferred to strains LC-E75 and FR-E01 by electroporation yielding > 10^8^ colonies, thus ensuring a ~1000X coverage of the library. Primers used for cloning are listed in S8 Table.

### High-throughput screens

The data for the screen performed with strain LC-E75 grown in rich medium was obtained from our previous study [26]. This screen was performed over 17 generations in triplicates from independent aliquots of the library generated from 3 independent transformations into strain LC-E75.

The phage screen was performed in triplicates as follows: FR-E01 was grown at 37°C from 1 mL aliquots stored at -80 °C into 500 mL LB. At OD_600_ = 0.2, dCas9 expression was induced by addition of 1 μM aTc (Acros Organics) to trigger the silencing of the target genes. The culture was grown to stationary phase (OD_600_ = 2) and diluted 100-fold in LB containing 1 μM aTc, 0.2% Maltose and 5 mM CaCl_2_. At OD_600_ = 0.4, 20 mL of the culture was sampled and the library was extracted by miniprep (Nucleospin Plasmid, Macherey-Nage) to obtain the sgRNA distribution before infection. The culture was then infected with 1 mL of λ, T4 or 186cI-ts stocks (10^7^ pfu / μL) to reach a MOI of 1 ensuring a high infection rate while limiting double infections. After 2 h at 37°C, the cultures were harvested (7 min– 4,000 g). Pellets were washed twice in PBS and the library was extracted by miniprep to obtain the sgRNA distribution after infection. For phage λ, the supernatant containing a mixture of λ and packaged library was collected and filtered (0.22 μm). Transduction was performed with 25 mL of lysate and 75 mL of stationary phase culture of MG1655 carrying λ prophage (providing resistance to super-infection) grown in LB supplemented with 0.2% maltose. After 30 min at 37°C, cells were harvested and resuspended in 1 mL LB and transduced cells were selected on 4 12x12 cm Petri dishes containing kanamycin. After 4h at 37°C, nascent colonies were washed and harvested in 5 mL LB-Kan. Each sample was split into 6 tubes from which library plasmids were extracted by miniprep (Nucleospin Plasmid, Macherey-Nage) before pooling back the 6 extractions into one sample for sequencing.

### Illumina sample preparation and sequencing

Library sequencing was performed as previously described [26]. Briefly, a customized Illumina sequencing method was designed to avoid problems arising from low library diversity when sequencing PCR products. Two Phusion PCR reactions (ThermoScientific) were used to generate the sequencing library with primers listed in S8 Table. The 1st PCR adds the 1st index. The 2nd PCR adds the 2nd index and flow cell attachment sequences. Sequencing is then performed using primer LC609 as a custom read 1 primer. Custom index primers were also used: LC499 reads index 1 and LC610 reads index 2. Sequencing was performed on a NextSeq 500 benchtop sequencer (Illumina). The first 2 cycles which read bases common to all clusters were set as dark cycles, followed by 20 cycles to read the guide. Using this strategy, we obtained on average 17 million reads per sample for the growth-based screen in LC-E75 and 4.6 million reads per sample for the phage screens in FR-E01 respectively.

### Data analysis

Raw sequencing files are available on the European Nucleotide Archive (https://www.ebi.ac.uk/ena) with the accession number PRJEB28256. Indexes were used to de-multiplex the data. Guides were filtered for potential off-target effect by discarding guides when the 9 PAM-proximal bases have a perfect match next to an NGG PAM in the promoter region of a gene (loosely defined as a window of 100bp before to 20bp after the start codon of all genes in the genome), or when the 11 PAM-proximal bases have a perfect match next to an NGG PAM allowing binding to the coding strand of a gene. Guides were also filtered to remove those carrying one of the 10 strongest bad seeds described in our previous study (AGGAA, TAGGA, ACCCA, TTGGA, TATAG, GAGGC, AAAGG, GGGAT, TAGAC and GTCCT) [26]. Guides with fewer than 20 reads in total were discarded.

Statistical analysis was performed from count data using the DESeq2 package [32] in R. This package allows comparison of expression data by modeling read counts using a negative binomial generalized linear model. The read counts of a non-targeting control guide RNA present in the library (5’-TGAGACCAGTCTAGGTCTCG-3’) were used as the normalization factor. A paired analysis was performed to compare each sample to its initial condition. The log2FoldChange (log2FC) value represents the enrichment or depletion of each sgRNA. For the phage screen, guides targeting the template strand of genes or outside of genes were excluded from the analysis as well as well guides with insufficient number of reads (BaseMean < 10), yielding a library of ~ 17,200 sgRNAs. The lists of all sgRNAs with computed log2FC values after the growth-based screen, after phage screens and after transduction assay are provided as S1, S4 and S6 Tables respectively. For each gene, the median log2FC value of the sgRNAs targeting the coding strand was used for ranking (S2 and S5 Tables). For the λ transduction experiment, a linear model was built for each gene to predict log2FC using the log2FC values of the essentiality screen and a Boolean factor considering the gene, or not, as a host factor. A given gene was regarded as a hit when the Boolean factor significantly improved the model after correction for multiple testing (ANOVA, FDR < 0.05), showing that the log2FC values obtained for this gene cannot be explained only by the fitness defect induced by the guides. The regression coefficient of this parameter termed as “estimate” was used for gene ranking. Genes whose silencing decreases the production of functional capsids have a negative estimate after transduction. A list of estimates and FDR for each gene is provided as S7 Table. Transriptional units (operons) were obtained from RegulonDB [90]. The log2FC values after the essentiality screen can be visualized on our web-based tool CRISPRbrowser (crispr.pasteur.fr). The code used for data analysis is available at gitlab.pasteur.fr/dbikard/dCas9_genome_wide_screen.

### Infection dynamics

sgRNAs were cloned into a psgRNAcos backbone through Golden Gate assembly using BsaI [91] and were electroporated into strain FR-E01. For essential gene *yrfF*, 4 mismatches were inserted at the 5’-end of the sgRNA to decrease expression of the target gene to intermediate levels. Constructions were validated by Sanger sequencing. The list of individual sgRNAs is provided in S9 Table. Strains were grown overnight and diluted 100-fold in LB medium containing 0.2% maltose, 1 μM aTc, 5 mM CaCl_2_ and kanamycin. At OD_600_ = 0.4, 90 μL of cultures were mixed in 96-well plates with 100 μL of fresh medium and 10 μL of a 8.10^4^ pfu/μL stock of the appropriate phage (MOI ~ 0.01). Infection dynamics were monitored in three replicates on an Infinite M200Pro (Tecan) at 37°C with shaking for 8h. Strains Δ*waaJ* and Δ*rcsB* were obtained from the Keio collection. The *rcsB* deletion was transferred to strain FR-E01 by P1 transduction.

### Gene deletions

Plasmid pKOBEG-A [92] carrying the λ-red system components under the control of an arabinose-inducible promoter was transformed into strain MG1655. A kanamycin resistance cassette was amplified by PCR with Phusion polymerase (ThermoScientific) from plasmid pKD4 with primers introducing homology regions with sequences flanking genes *alsK*, *bcsB* or *entD*. Electrocompetent MG1655-pKOBEG-A cells were prepared with arabinose (Sigma) before transformation of 1 μg of the DNA fragments. Recovery and overnight incubation were performed at 30°C with arabinose and kanamycin. The next day, colonies were restreaked and incubated at 37°C. Primers used for pKD4 amplification and colony screening are provided in S8 Table.

### RT-qPCR

Overnight cultures were diluted 1:100 in 3 mL LB containing 1 μM aTc. Cells were further grown for 2h before RNA extraction using Direct-zol RNA MiniPrep (Zymo Research) followed by DNAse treatment using TURBO DNA-free Kit (Thermo Fisher Scientific). All RNAs were reverse transcribed into cDNA using the Transcriptor First Strand cDNA Synthesis Kit (Roche) using 500 ng RNA. qPCR was performed with the FastStart Essential DNA Green master mix (Roche) in a LightCycler 96 (Roche) following the manufacturer’s instructions. qPCR was performed in two technical replicates and three biological replicates. Relative gene expression was computed on LightCycler 96 software (Roche) using the ΔΔCq method [93] after normalization by 5S rRNA (*rrsA*). qPCR primers are listed in S10 Table.

## Supporting information

S1 Fig Target regions of guides that produce a strong fitness defect while targeting multiple positions. (A) 348/1932 sgRNAs that simultaneously target several positions induce a fitness defect (log2FC < -2). These guides mostly target rRNAs, tRNAs, repeat regions or elongation factor genes (tufA-tufB). (B) Among sgRNAs targeting REP elements, sgRNAs simultaneously targeting several regions have more chance of inducing a fitness defect than sgRNAs targeting a single region (Fisher’s exact test, odds ratio = 0.33, p < 2.10–5). (C) Example of a sgRNA simultaneously targeting 3 repeat regions. sgRNAs targeting the +1 or -1 strand are dotted in red or blue respectively. Repeat regions are represented as grey boxes.
(PNG)

S2 Fig Gene interaction network of the top 100 genes having the lowest median log2FoldChange. The STRING database was used to compute a gene interaction network [94]. Genes were colored by function. Line thickness indicates confidence of the interaction.
(PNG)

S3 Fig sgRNAs with off-targets in essential genes induce a strong fitness defect. (**A**) A sgRNA targeting *mdfA* has a 10-nt perfect match to *rpsL*. (**B**) A sgRNA targeting *flgC* has a 10-nt perfect match to *lptG*. (**A,B**) Matched base pairs are shown in blue. On the plots, sgRNAs targeting the +1 strand or the -1 strand are shown in red or blue respectively. A black arrow indicates the sgRNA with an off-target activity. A dashed line indicates the off-target position.
(PNG)

S4 Fig Predicted internal promoters within operons. We identified 7 operons in which an expected polar effect is not observed, i.e guides targeting a gene upstream of an essential or near-essential gene are not depleted. This suggests that the downstream gene can be expressed from an internal promoter. Promoters predicted from a recent transcription start site dataset are shown as dashed arrow [38].
(PNG)

S5 Fig Replacement of genes *alsK*, *bcsB* and *entD* by a Kanamycin resistance cassette through λ-red recombination shows that these genes are not essential. The kanamycin resistance cassette from plasmid pKD4 was amplified with primers designed to introduce 50 bp-long homologies with regions flanking genes *alsK*, *bcsB* and *entD*. Primer couples P1 + P2, P1’ + P2’ and P1” + P2” were designed to flank the genetic region of *alsK*, *bcsB* and *entD* respectively, while primer couples P3 + P4, P3’ + P4’ and P3” + P4” were designed to amplify within *alsK*, *bcsB* and *entD* respectively to demonstrate that gene duplication did not occur during the experiment.
(PNG)

S6 Fig Inefficient dCas9-mediated repression of genes regulated by negative feedback loops. *lexA* and *rho* are two well-known essential genes whose product inhibits their own expression and which are classified as nonessential in our screen. (**A**) Relative *lexA* or *rho* expression was measured in presence of a *lexA*- or *rho*-targeted sgRNA respectively, with or without dCas9 repression (± aTc), showing a low repression activity (66.4% and 85.2% respectively). RT-qPCR results are shown for 3 biological replicates and 2 technical replicates. (**B**) To measure the activity of the *lexA* and rho promoters while targeting the respective gene with dCas9, we built plasmids pFR42 which expresses sfGFP from the *lexA* promoter and pFR43 which expresses sfGFP from the *rho* promoter. These plasmids also express the corresponding sgRNA constitutively. (**C**) pFR42 and pFR43 were transformed into strain LC-E75 used in the screen. An overnight culture was diluted 100-fold with or without aTc and OD600 and GFP fluorescence were measured overtime. Raw GFP fluorescence after 12h was normalized by OD600 and the normalized fluorescence of the control (LC-E75 with psgRNAcos) was subtracted. Bar plot shows mean ± standard deviation (n = 3).
(PNG)

S7 Fig Unexpected fitness defects shed light on atypical gene organizations. (**A**) *yejL* is located upstream of the sensitive essential gene *yejM*. (**B**) *ypaB* contains the promoter of the essential gene *nrdA*. (**C**) *sroG* is a riboswitch controlling the expression of the essential gene *ribB*. (**D**) *yceQ* is annotated as essential but is actually located upstream *rne* in the opposite direction and contains the promoter driving the expression of *rne*. (**E**) *psrO* encodes a small RNA and is located in the promoter region of near-essential gene *pnp*. sgRNAs targeting the +1 or -1 strand are shown as red or blue dots respectively.
(PNG)

S8 Fig Strains LC-E75 and FR-E01 produce similar concentrations of dCas9. Strains LC-E75 and FR-E01 were grown overnight and diluted 1:100 with aTc for 2 h before harvesting. Samples were run in NuPAGE Novex Bis-Tris gels in reducing condition before transfer to PVDF membranes. Rabbit monoclonal CRISPR-Cas9 antibody and rabbit polyclonal RecA antibody were used.
(PNG)

S9 Fig Phage λ can package plasmid psgRNAcos upon infection. (**A**) MG1655 carrying psgRNAcos was infected with λ at MOI = 1. The lysate containing a mix of phage and packaged cosmid was extracted after 2 h and the relative concentrations of phage and cosmid were measured by plaque assay and by transduction into strain MG1655::λ respectively. This strain carries the λ lysogen and is thus resistant to superinfection by the λ particles present in the lysate. (**B,C**) Bar plot and dot plot show mean ± standard deviation (n = 9).
(PNG)

S10 Fig sgRNAs in the library with a perfect match in λ phage genome. sgRNAs targeting the positive or negative strand are dotted in red or blue respectively. Genes colored in yellow, orange and red respectively correspond to early right, early left and late operon.
(PNG)

S1 Table List of sgRNAs with log2FC values after the growth-based screen in strain LC-E75. Each sgRNA is indexed with its position, orientation (ori), target strand (coding) and computed fold change (log2FC, padj and gamma). Information on the targeted gene is also provided: name (gene), essentiality (essential), position (gene_left, gene_right) and orientation (gene_ori).
(CSV)

S2 Table List of genes with median log2FC values after growth-based screen. The following information is provided. Gene name (gene), essentiality (essential), orientation (gene_ori), position (gene_left, gene_right), median log2FC for each strand (median_coding and median_template) and median absolute deviation (mad_coding and mad_template), number of guides targeting the coding or template strand (coding and template), as well as information on operon structure (operon). This table contains all genes targeted in the screen including protein-coding and RNAs.
(CSV)

S3 Table List of essential genes not found essential in our screen. (XLSX)

S4 Table List of sgRNAs with log2FC values after phage screens. Each sgRNA is indexed with its position, orientation (ori), strand (coding), information on the targeted gene (gene, essential, gene_left, gene_right, gene_ori) and computed fold change for each phage (log2FC_lambda, log2FC_T4 and log2FC_186).
(CSV)

S5 Table List of genes with resistance scores after phage screens. Each gene is indexed with its information (gene, essential), median log2FC (Resistance_Score_lambda, Resistance_Score_T4 and Resistance_Score_186) and median absolute deviation of guides targeting the coding stand for each phage, number of guides targeting the coding strand used for calculations (sgRNAs), as well as information on operon structures (operon).
(CSV)

S6 Table List of sgRNAs with log2FC values after transduction screen. Each sgRNA is indexed with its position, orientation (ori), strand (coding), information on the targeted gene (gene, essential, gene_left, gene_right, gene_ori) and computed fold change after transduction (log2FC).
(CSV)

S7 Table List of genes with estimates and FDR after transduction screen. Each gene is indexed with its information (gene, essential), operon information (operon) and number of guides targeting the coding strand used for calculations (sgRNAs). For each gene, a linear model was built to take the effect on cell fitness into account (see Methods). The regression coefficient of the Boolean parameter termed as “estimate” is used for gene ranking. Genes whose silencing decreases the production of functional capsids have a negative estimate after transduction. P-value and FDR associated to this parameter are also displayed for each gene.
(CSV)

S8 Table List of primers used for cloning and sequencing. (DOCX)

S9 Table List of individual sgRNAs used in this study. (DOCX)

S10 Table List of primers used for qPCR. (DOCX)
