# Supplementary figures and images for: Genome-wide CRISPR-dCas9 screens in E. coli identify essential genes and phage host factors
Source: PLoS Genet. 2018 Nov 7;14(11):e1007749. doi: 10.1371/journal.pgen.1007749 (PMC6242692; doi:10.1371/journal.pgen.1007749)

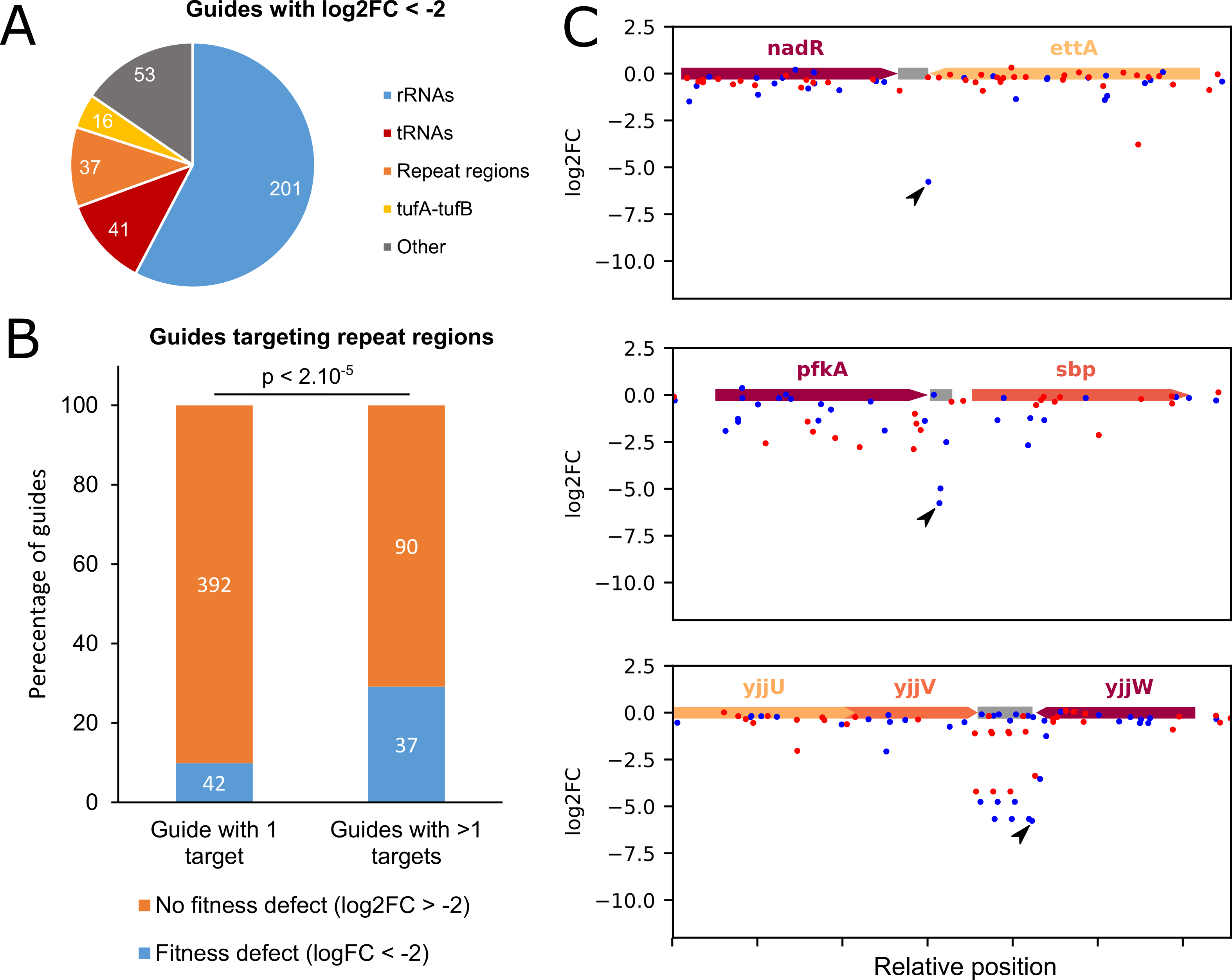

Supplement: S1 Fig — (A) 348/1932 sgRNAs that simultaneously target several positions induce a fitness defect (log2FC < -2). These guides mostly target rRNAs, tRNAs, repeat regions or elongation factor genes (tufA-tufB). (B) Among sgRNAs targeting REP elements, sgRNAs simultaneously targeting several regions have more chance of inducing a fitness defect than sgRNAs targeting a single region (Fisher’s exact test, odds ratio = 0.33, p < 2.10–5). (C) Example of a sgRNA simultaneously targeting 3 repeat regions. sgRNAs targeting the +1 or -1 strand are dotted in red or blue respectively. Repeat regions are represented as grey boxes. (PNG) [file pgen.1007749.s001.png]

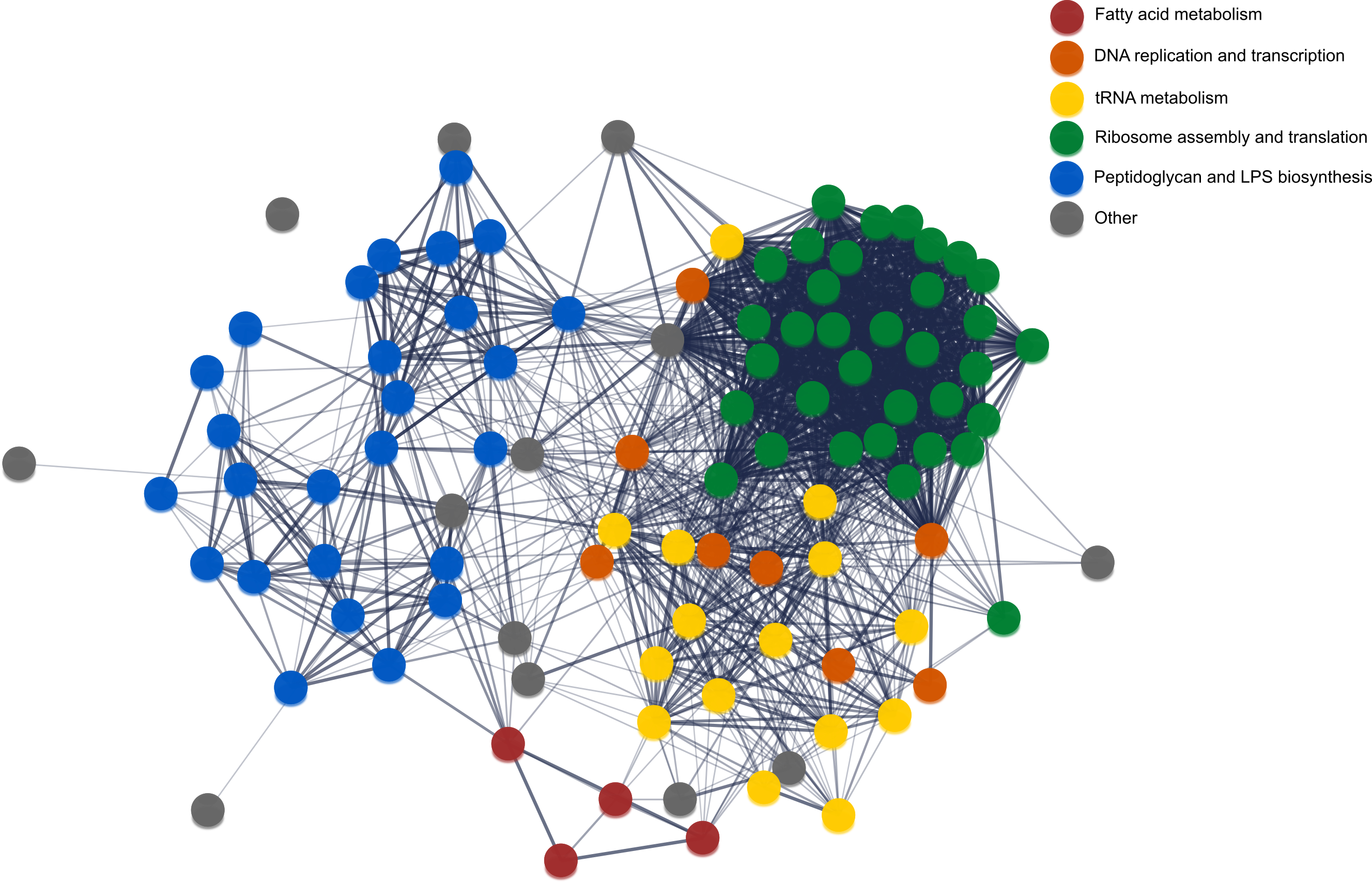

Supplement: S2 Fig — The STRING database was used to compute a gene interaction network [94]. Genes were colored by function. Line thickness indicates confidence of the interaction. (PNG) [file pgen.1007749.s002.png]

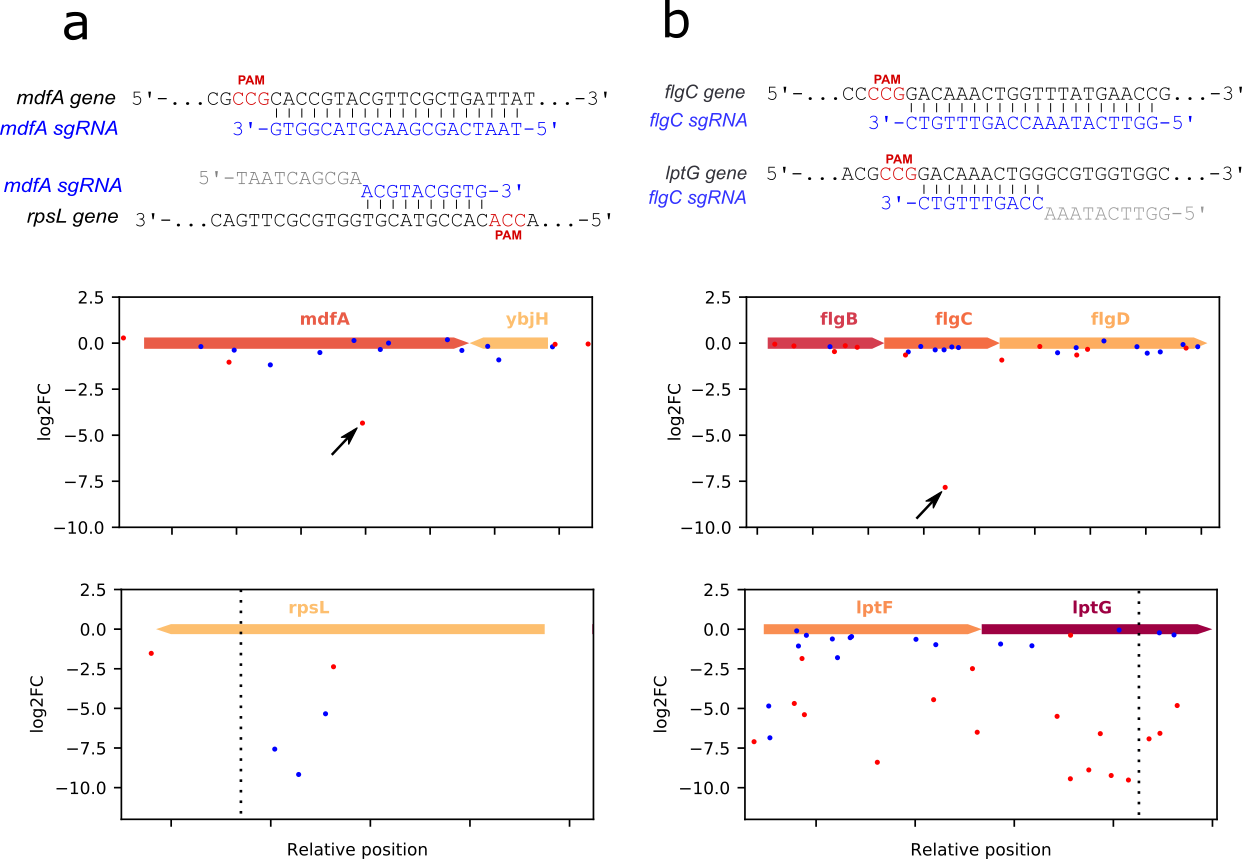

Supplement: S3 Fig — (A) A sgRNA targeting mdfA has a 10-nt perfect match to rpsL. (B) A sgRNA targeting flgC has a 10-nt perfect match to lptG. (A,B) Matched base pairs are shown in blue. On the plots, sgRNAs targeting the +1 strand or the -1 strand are shown in red or blue respectively. A black arrow indicates the sgRNA with an off-target activity. A dashed line indicates the off-target position. (PNG) [file pgen.1007749.s003.png]

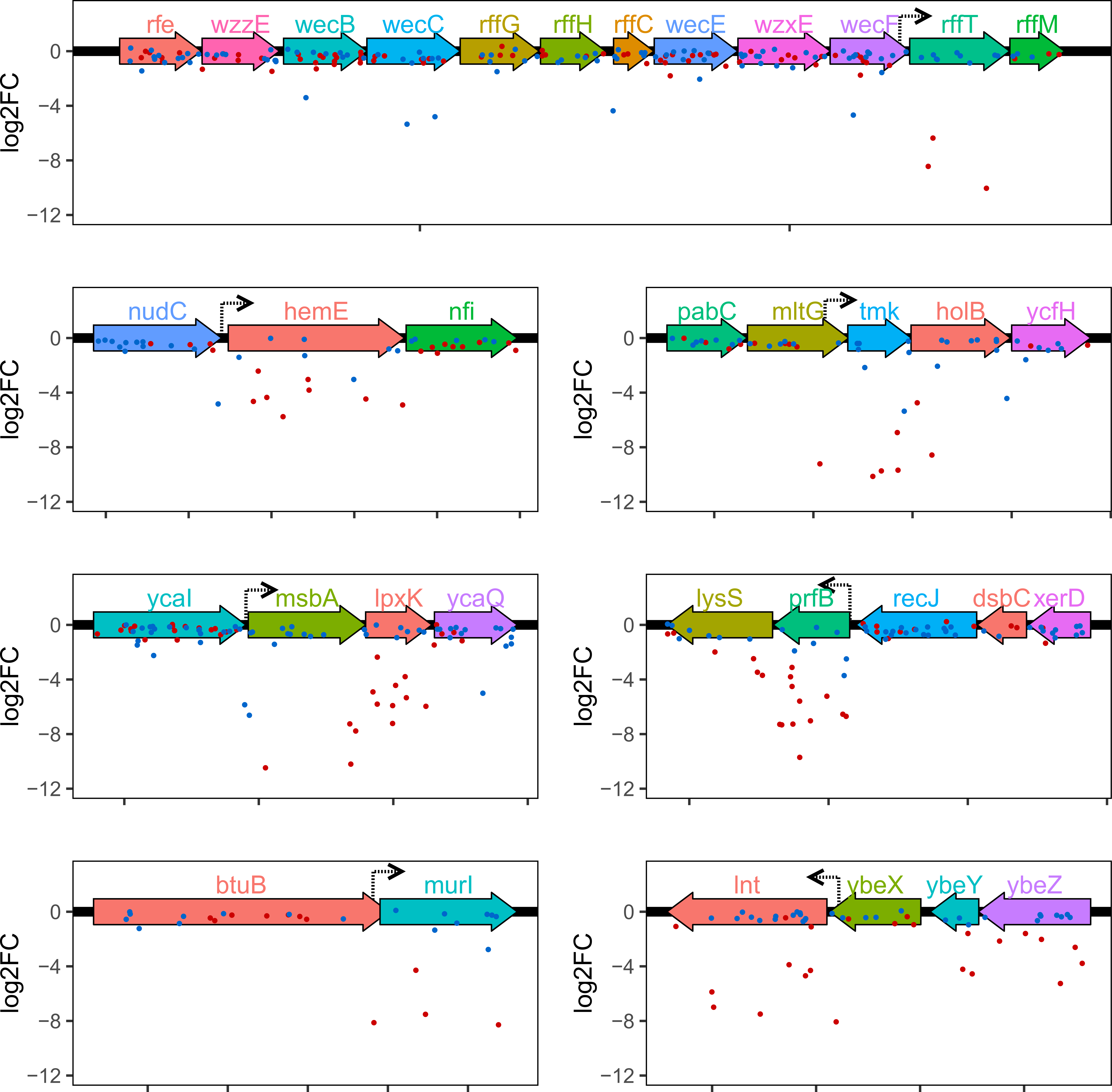

Supplement: S4 Fig — We identified 7 operons in which an expected polar effect is not observed, i.e guides targeting a gene upstream of an essential or near-essential gene are not depleted. This suggests that the downstream gene can be expressed from an internal promoter. Promoters predicted from a recent transcription start site dataset are shown as dashed arrow [38]. (PNG) [file pgen.1007749.s004.png]

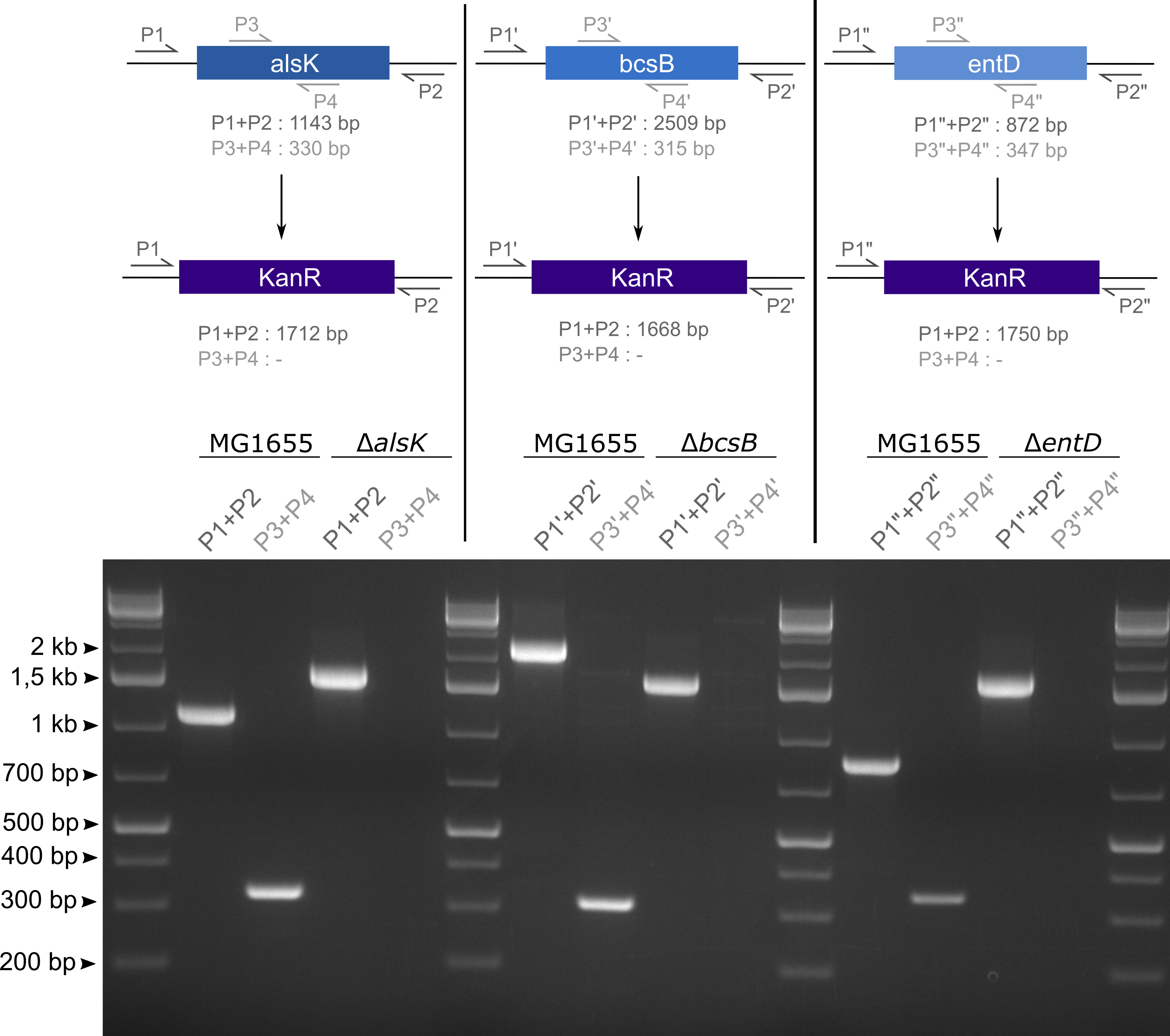

Supplement: S5 Fig — The kanamycin resistance cassette from plasmid pKD4 was amplified with primers designed to introduce 50 bp-long homologies with regions flanking genes alsK, bcsB and entD. Primer couples P1 + P2, P1’ + P2’ and P1” + P2” were designed to flank the genetic region of alsK, bcsB and entD respectively, while primer couples P3 + P4, P3’ + P4’ and P3” + P4” were designed to amplify within alsK, bcsB and entD respectively to demonstrate that gene duplication did not occur during the experiment. (PNG) [file pgen.1007749.s005.png]

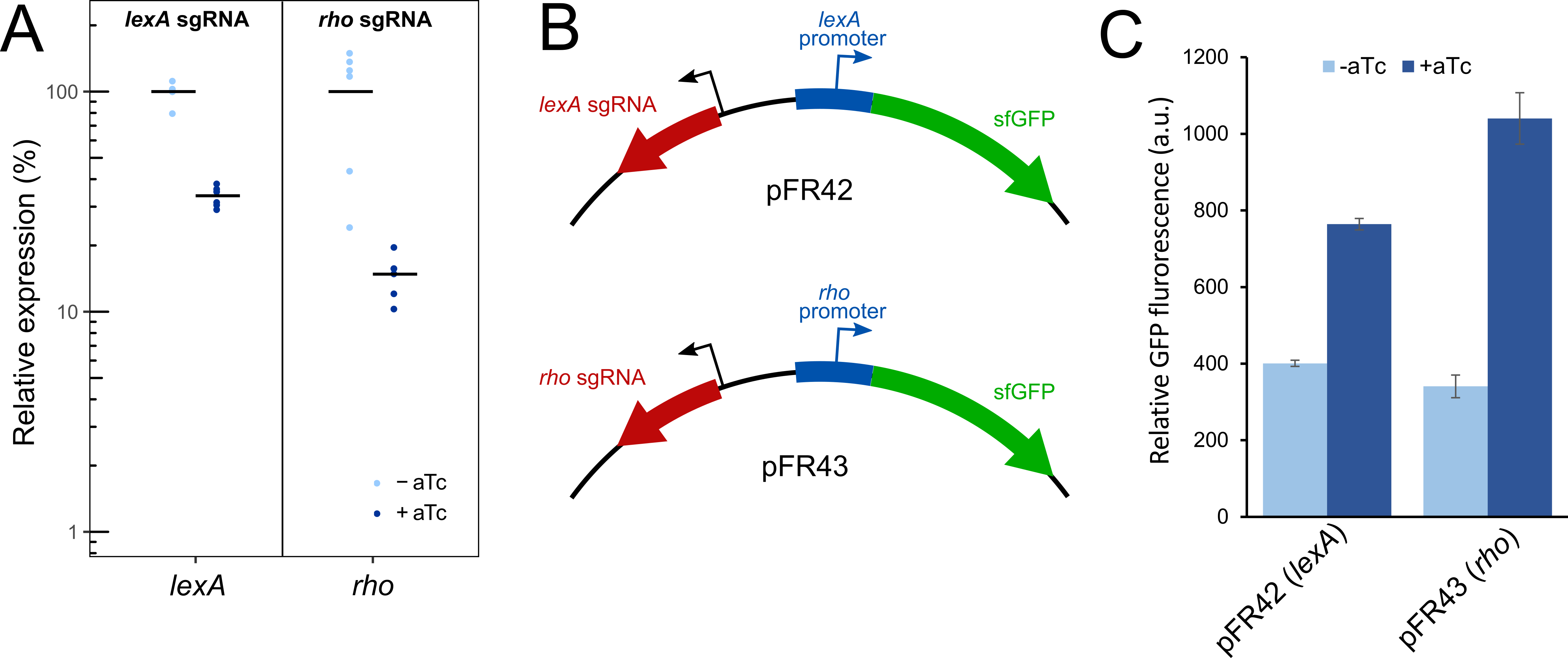

Supplement: S6 Fig — lexA and rho are two well-known essential genes whose product inhibits their own expression and which are classified as nonessential in our screen. (A) Relative lexA or rho expression was measured in presence of a lexA- or rho-targeted sgRNA respectively, with or without dCas9 repression (± aTc), showing a low repression activity (66.4% and 85.2% respectively). RT-qPCR results are shown for 3 biological replicates and 2 technical replicates. (B) To measure the activity of the lexA and rho promoters while targeting the respective gene with dCas9, we built plasmids pFR42 which expresses sfGFP from the lexA promoter and pFR43 which expresses sfGFP from the rho promoter. These plasmids also express the corresponding sgRNA constitutively. (C) pFR42 and pFR43 were transformed into strain LC-E75 used in the screen. An overnight culture was diluted 100-fold with or without aTc and OD600 and GFP fluorescence were measured overtime. Raw GFP fluorescence after 12h was normalized by OD600 and the normalized fluorescence of the control (LC-E75 with psgRNAcos) was subtracted. Bar plot shows mean ± standard deviation (n = 3). (PNG) [file pgen.1007749.s006.png]

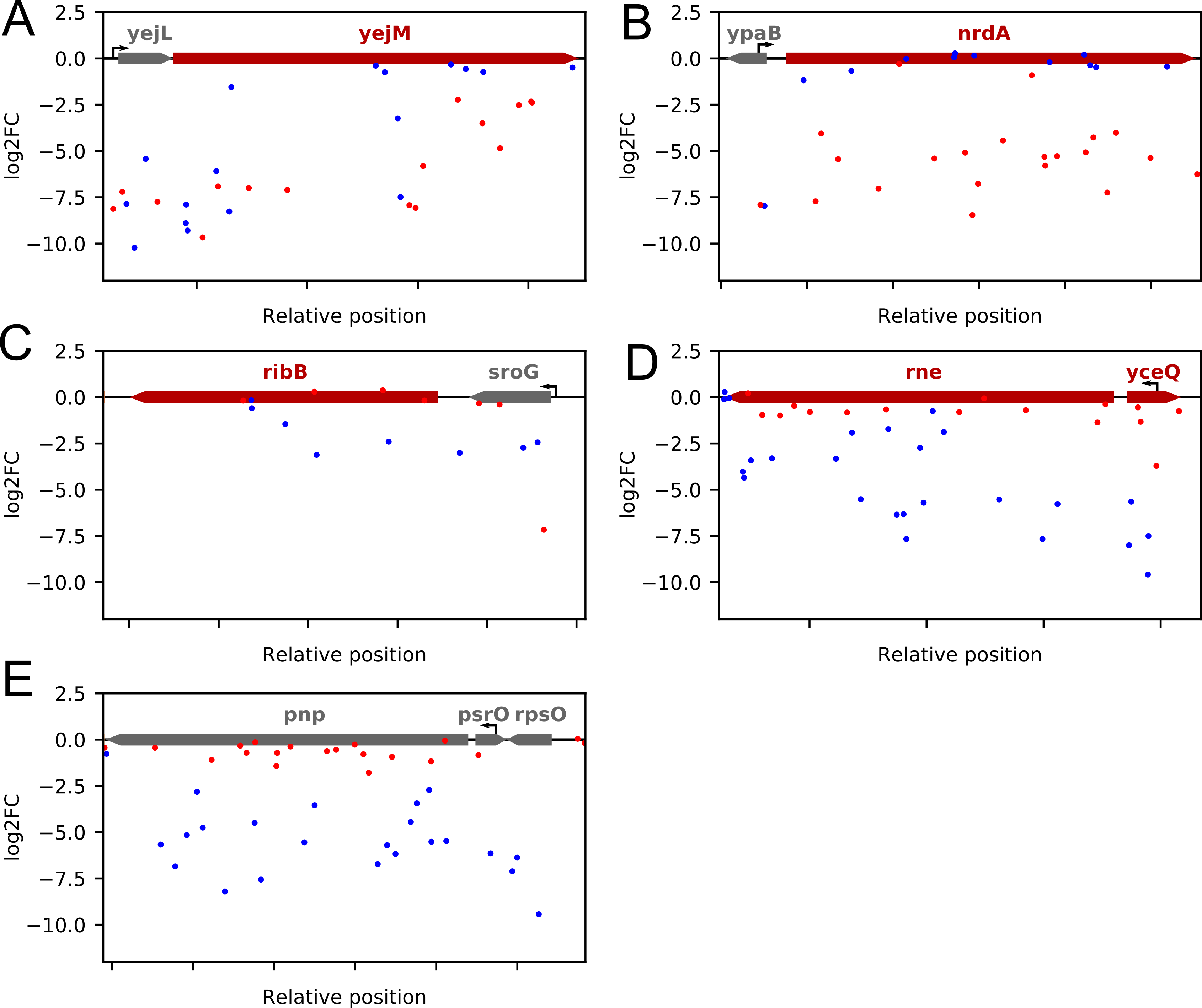

Supplement: S7 Fig — (A) yejL is located upstream of the sensitive essential gene yejM. (B) ypaB contains the promoter of the essential gene nrdA. (C) sroG is a riboswitch controlling the expression of the essential gene ribB. (D) yceQ is annotated as essential but is actually located upstream rne in the opposite direction and contains the promoter driving the expression of rne. (E) psrO encodes a small RNA and is located in the promoter region of near-essential gene pnp. sgRNAs targeting the +1 or -1 strand are shown as red or blue dots respectively. (PNG) [file pgen.1007749.s007.png]

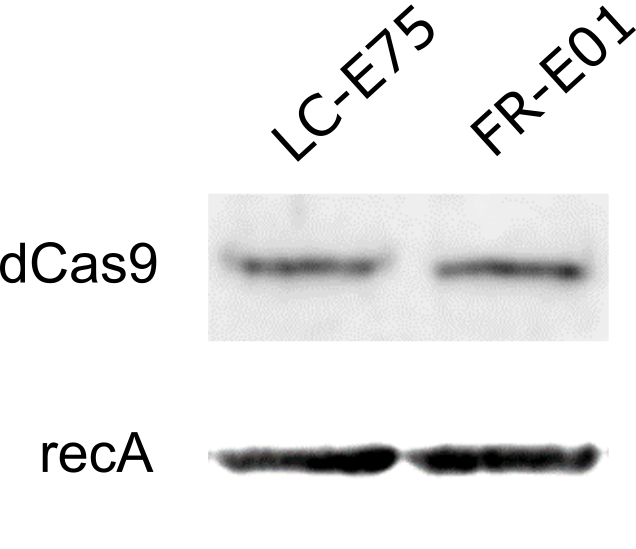

Supplement: S8 Fig — Strains LC-E75 and FR-E01 were grown overnight and diluted 1:100 with aTc for 2 h before harvesting. Samples were run in NuPAGE Novex Bis-Tris gels in reducing condition before transfer to PVDF membranes. Rabbit monoclonal CRISPR-Cas9 antibody and rabbit polyclonal RecA antibody were used. (PNG) [file pgen.1007749.s008.png]

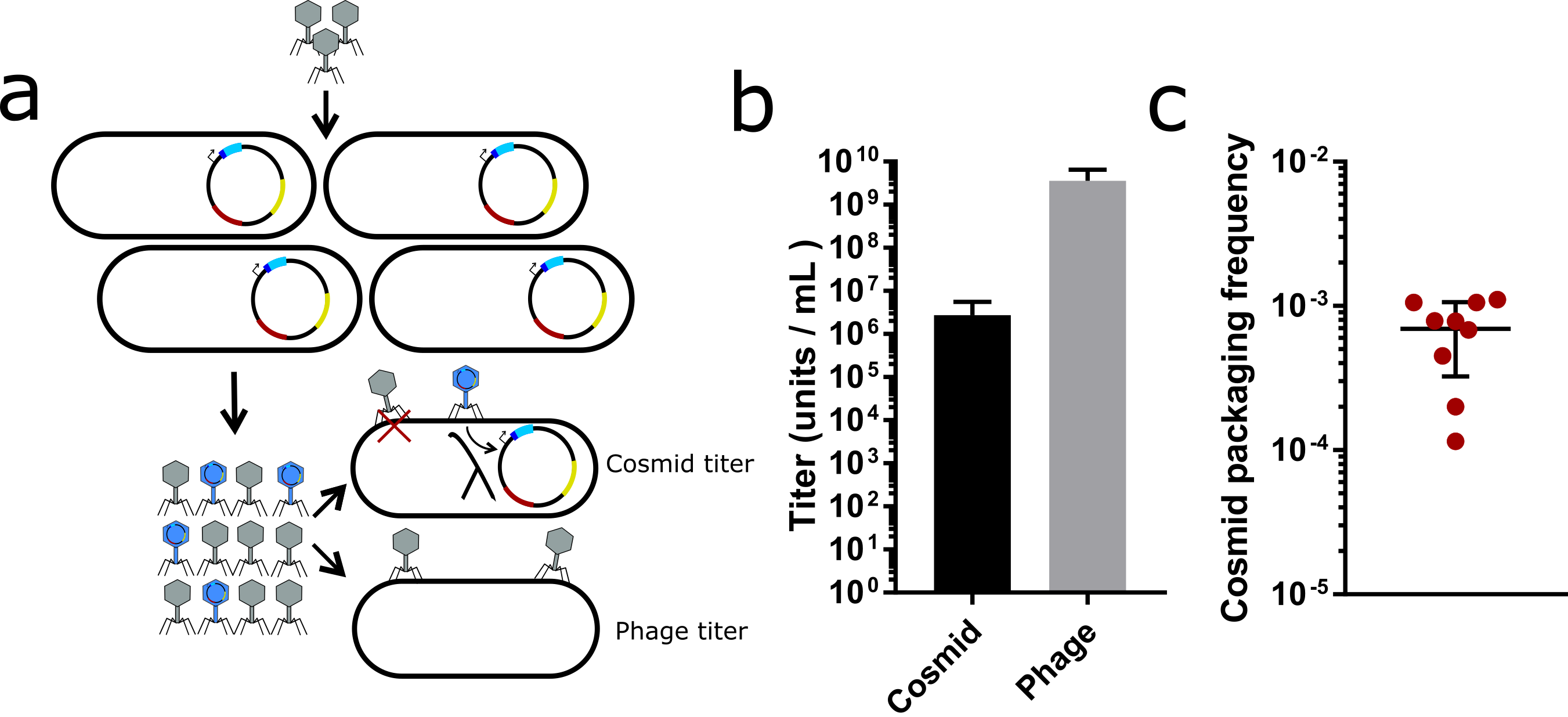

Supplement: S9 Fig — (A) MG1655 carrying psgRNAcos was infected with λ at MOI = 1. The lysate containing a mix of phage and packaged cosmid was extracted after 2 h and the relative concentrations of phage and cosmid were measured by plaque assay and by transduction into strain MG1655::λ respectively. This strain carries the λ lysogen and is thus resistant to superinfection by the λ particles present in the lysate. (B,C) Bar plot and dot plot show mean ± standard deviation (n = 9). (PNG) [file pgen.1007749.s009.png]

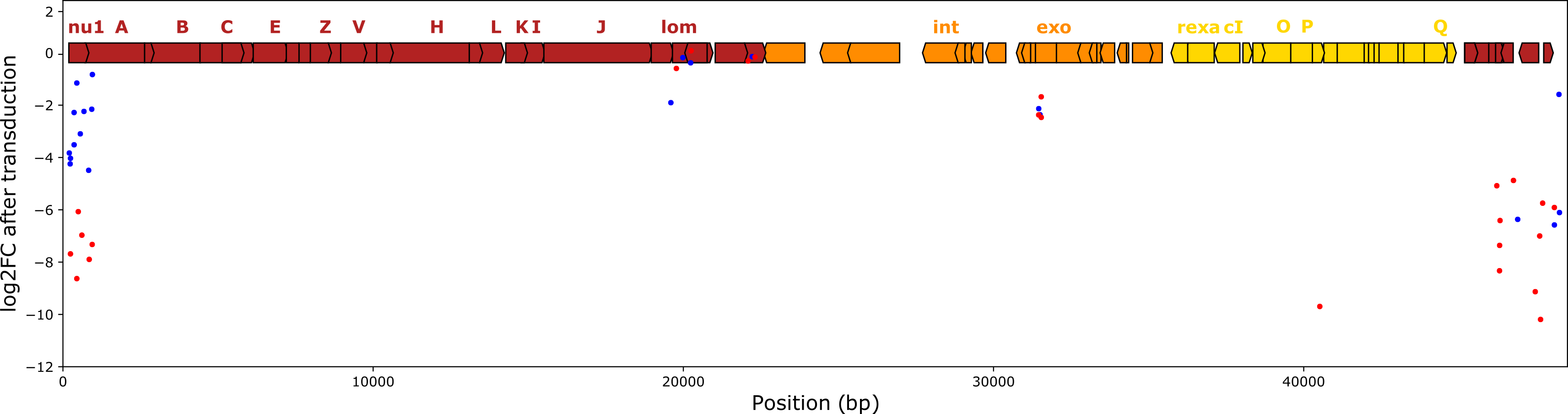

Supplement: S10 Fig — sgRNAs targeting the positive or negative strand are dotted in red or blue respectively. Genes colored in yellow, orange and red respectively correspond to early right, early left and late operon. (PNG) [file pgen.1007749.s010.png]
