# Supplementary material for: Genome-wide CRISPR-dCas9 screens in E. coli identify essential genes and phage host factors
Source: PLoS Genet. 2018 Nov 7;14(11):e1007749. doi: 10.1371/journal.pgen.1007749 (PMC6242692; doi:10.1371/journal.pgen.1007749)
Supplement: S8 Table — (DOCX) [file pgen.1007749.s018.docx]

| **Supplemental Table S8 \| List of primers used for cloning and sequencing** | | | |
| --- | --- | --- | --- |
|  | |  | |
| **Name** | **Role** | | **Sequence (5’-3’)** |
| LC81 | Verification of dCas9 construct integration | | ACTTAACGGCTGACATGG |
| LC82 | Verification of dCas9 construct integration | | ACGAGTATCGAGATGGCA |
| LC85 | Verification of dCas9 construct integration | | GGAATCAATGCCTGAGTG |
| LC86 | Verification of dCas9 construct integration | | GGCATCAACAGCACATTC |
| LC124 | Amplification of dCas9 insert | | CTGCCAGGAATTGGGGATC |
| LC125 | Amplification of dCas9 insert | | CAGTTTAGGTTAGGCGCCAT |
| LC293 | Amplification of psgRNAcos backbone | | GTTTTAGAGCTAGAAATAGCAAGTTAA |
| LC294 | Amplification of psgRNAcos backbone | | ACTAGTATTATACCTAGGACTGAGCTA |
| LC296 | Amplification of oligos library | | TATATTTTAGGAATTCTAAAGATCTTTGACAGCTAGCTCAGTCCTAGGTATAATACTAGT |
| LC297 | Amplification of oligos library | | ACTTTTTCAAGTTGATAACGGACTAGCCTTATTTTAACTTGCTATTTCTAGCTCTAAAAC |
| LC606 | Index 1 for custom sample preparation | | TTCCCTACACGACGCTCTTCCGATCTTAGANNNNGCACGCCCGTCGCTCAGTCCTAGGTATAATACTA |
| LC607 | Index 1 for custom sample preparation | | TTCCCTACACGACGCTCTTCCGATCTCTCTNNNNGCACGCCCGTCGCTCAGTCCTAGGTATAATACTA |
| LC608 | Index 1 for custom sample preparation | | TTCCCTACACGACGCTCTTCCGATCTATTCNNNNGCACGCCCGTCGCTCAGTCCTAGGTATAATACTA |
| LC863 | Reverse primer for sequencing PCR1 | | GTGACTGGAGTTCAGACGTGTGCTCTTCCGATCTNNNNNNAAAGGACCCGTAAAGTGATAATGAT |
| LC418 | Index 2 for custom sample preparation | | CAAGCAGAAGACGGCATACGAGATAATGAGCGGTGACTGGAGTTCAGACG |
| LC419 | Index 2 for custom sample preparation | | CAAGCAGAAGACGGCATACGAGATGGAATCTCGTGACTGGAGTTCAGACG |
| LC420 | Index 2 for custom sample preparation | | CAAGCAGAAGACGGCATACGAGATTTCTGAATGTGACTGGAGTTCAGACG |
| LC421 | Index 2 for custom sample preparation | | CAAGCAGAAGACGGCATACGAGATACGAATTCGTGACTGGAGTTCAGACG |
| LC422 | Index 2 for custom sample preparation | | CAAGCAGAAGACGGCATACGAGATAGCTTCAGGTGACTGGAGTTCAGACG |
| LC423 | Index 2 for custom sample preparation | | CAAGCAGAAGACGGCATACGAGATGCGCATTAGTGACTGGAGTTCAGACG |
| LC578 | Index 2 for custom sample preparation | | CAAGCAGAAGACGGCATACGAGATTTCGCGGAGTGACTGGAGTTCAGACG |
| LC579 | Index 2 for custom sample preparation | | CAAGCAGAAGACGGCATACGAGATGCGCGAGAGTGACTGGAGTTCAGACG |
| LC580 | Index 2 for custom sample preparation | | CAAGCAGAAGACGGCATACGAGATCTATCGCTGTGACTGGAGTTCAGACG |
| LC415 | Forward primer for sequencing PCR2 | | AATGATACGGCGACCACCGAGATCTACACTCTTTCCCTACACGACGCT |
| LC609 | Custom read 1 primer for sequencing | | GCACGCCCGTCGCTCAGTCCTAGGTATAATACTA |
| LC499 | Index primer 1 for sequencing | | GATCGGAAGAGCACACGTCTGAACTCCAGTCAC |
| LC610 | Index primer 2 for sequencing | | TATTATACCTAGGACTGAGCGACGGGCGTGC |
| FR126 | pKD4 amplification for *alsK* deletion | | GAGAATTATGACCGCGCAGATTCTGGCTGCAAAAAGCGAGGTACAGCCTCTGTGTAGGCTGGAGCTGCTTC |
| FR127 | pKD4 amplification for *alsK* deletion | | TACCTGTCGTTGGCACAGCGGGACATTGCGCTACTGTGCCAACGTTTTACATGGGAATTAGCCATGGTCC |
| FR128 | pKD4 amplification for *bcsB* deletion | | AACGGCACAACCATCGGATCAGGCTTTGGCTCAACAATGATGATAACGCGTGTGTAGGCTGGAGCTGCTTC |
| FR129 | pKD4 amplification for *bcsB* deletion | | GCCAGCAGCAGCATCGTCACGATTCCACTACGCAACACATTCATCTTCAAATGGGAATTAGCCATGGTCC |
| FR130 | pKD4 amplification for *entD* deletion | | TAGCGCATCAGGCAGTTTTGCGTTTGTCATCAGTCTCGAATATGGTCGATTGTGTAGGCTGGAGCTGCTTC |
| FR131 | pKD4 amplification for *entD* deletion | | TCGCCCGTGGTCAGTGATGGCTGCGGGCGAATCGTACCAGATGTTGTCAAATGGGAATTAGCCATGGTCC |
| FR134 | Screening for *alsK* deletion (P1) | | TAGGCATTTGTGCTGAAGAGGG |
| FR135 | Screening for *alsK* deletion (P2) | | GCGGAAAATATCGACGAAGCAT |
| FR136 | Screening for *bcsB* deletion (P1’) | | AACACTAAAGGCAGCCAGCA |
| FR137 | Screening for *bcsB* deletion (P2’) | | ACTTCTCTGGTTTCCTGGGT |
| FR138 | Screening for *entD* deletion (P1”) | | TCCGGTTGTCAGGTCAGGAT |
| FR139 | Screening for *entD* deletion (P2”) | | TTGACAAAGTGCGCGTCGTT |
| FR157 | Screening for *alsK* deletion (P3) | | ATATCCATCACGCCACCG |
| FR158 | Screening for *alsK* deletion (P4) | | GGGGATGGGGTTCGCA |
| FR159 | Screening for *bcsB* deletion (P3’) | | GATAGCCGCCAGCACC |
| FR160 | Screening for *bcsB* deletion (P4’) | | GCAGAAACCCGGTCAACG |
| FR161 | Screening for *entD* deletion (P3”) | | TGCGCTCTCTTTGGCG |
| FR162 | Screening for *entD* deletion (P4”) | | TGCAACACGCTGGACG |
|  |  | |  |
