# Supplementary material for: Genome-wide CRISPR-dCas9 screens in E. coli identify essential genes and phage host factors
Source: PLoS Genet. 2018 Nov 7;14(11):e1007749. doi: 10.1371/journal.pgen.1007749 (PMC6242692; doi:10.1371/journal.pgen.1007749)
Supplement: S9 Table — (DOCX) [file pgen.1007749.s019.docx]

| **Supplemental Table S9 \| List of individual sgRNAs used in this study** | | | |
| --- | --- | --- | --- |
| **Name** | **Target sequence** | **Forward primer** | **Reverse primer** |
| nusG | CCGATGAAGCCCATCACACG | TAGTCCGATGAAGCCCATCACACG | AAACCGTGTGATGGGCTTCATCGG |
| yrfF-4m | ATCCGCGAAGGCGTTGGTCC | TAGTTAGGGCGAAGGCGTTGGTCC | AAACGGACCAACGCCTTCGCCCTA |
| gyrA1-T | GCGCTGCCAGATGTCCGAGA | TAGTGCGCTGCCAGATGTCCGAGA | AAACTCTCGGACATCTGGCAGCGC |
| gyrA2-T | TGTTCGTCACCGCCGTGAAG | TAGTTGTTCGTCACCGCCGTGAAG | AAACCTTCACGGCGGTGACGAACA |
| gyrA3-T | GTTGCTAATCCGTGGCAGCT | TAGTGTTGCTAATCCGTGGCAGCT | AAACAGCTGCCACGGATTAGCAAC |
| glyQ1-T | ATGACCTGTCTGCGCGAGCT | TAGTATGACCTGTCTGCGCGAGCT | AAACAGCTCGCGCAGACAGGTCAT |
| glyQ2-T | CCGTTTCGTGGAAGATAACT | TAGTCCGTTTCGTGGAAGATAACT | AAACAGTTATCTTCCACGAAACGG |
| glyQ3-T | TAACTGGGAAAACCCGACGC | TAGTTAACTGGGAAAACCCGACGC | AAACGCGTCGGGTTTTCCCAGTTA |
| lexA | AGATGTTCTTCAGCCGCGTT | TAGTAGATGTTCTTCAGCCGCGTT | AAACAACGCGGCTGAAGAACATCT |
| rho | GCGGGGTTAAGTTCTCAAAG | TAGTGCGGGGTTAAGTTCTCAAAG | AAACCTTTGAGAACTTAACCCCGC |
