# Supplementary material for: Genome-wide CRISPR-dCas9 screens in E. coli identify essential genes and phage host factors
Source: PLoS Genet. 2018 Nov 7;14(11):e1007749. doi: 10.1371/journal.pgen.1007749 (PMC6242692; doi:10.1371/journal.pgen.1007749)
Supplement: S10 Table — (DOCX) [file pgen.1007749.s020.docx]

| **Supplemental Table S10 \| List of primers used for qPCR** | | |
| --- | --- | --- |
| **Name** | **Role** | **Sequence (5’-3’)** |
| BG208 | Forward *gyrA* primer | CTGCGTTTGCAGAAACTGAC |
| BG209 | Reverse *gyrA* primer | ATCGGCGCTACCAAGAATAC |
| LC776 | Forward *glyQ* primer | AGATCACCTACGGTCTGGAA |
| LC777 | Reverse *glyQ* primer | TGCTCCACTTCGTTCTGATG |
| FR86 | Forward *wza* primer | CGTTGATGGCGTCGAGAATA |
| FR87 | Reverse *wza* primer | CTCCGGTCAGGTGAATAAATCC |
| FR88 | Forward *wzb* primer | TACCTGCTCTGCGTTCAATG |
| FR89 | Reverse *wzb* primer | GCAGCGGTGTACACATTACT |
| FR100 | Forward *lexA* primer | GGCGCAACAGCATATTGAAG |
| FR101 | Reverse *lexA* primer | GCAGCAGGAAATCAGCATTC |
| FR104 | Forward *rho* primer | GGTACAGGCAACATGGAACT |
| FR105 | Reverse *rho* primer | CCAGAACGGTTGTAGTCGATAG |
| LC740 | Forward *rrsA* primer | CTAATCCCATCTGGGCACATC |
| LC741 | Reverse *rrsA* primer | CTAATCCCATCTGGGCACATC |
